# Constraining global transport of perfluoroalkyl acids on sea spray aerosol using field measurements

**DOI:** 10.1126/sciadv.adl1026

**Published:** 2024-04-05

**Authors:** Bo Sha, Jana H. Johansson, Matthew E. Salter, Sara M. Blichner, Ian T. Cousins

**Affiliations:** ^1^ Department of Environmental Science, Stockholm University, Stockholm, Sweden.; ^2^ Department of Thematic Studies—Environmental Change, Linköping University, Linköping, Sweden.; ^3^ Bolin Centre for Climate Research, Stockholm, Sweden.

## Abstract

Perfluoroalkyl acids (PFAAs) are highly persistent anthropogenic pollutants that have been detected in the global oceans. Our previous laboratory studies demonstrated that PFAAs in seawater are remobilized to the air in sea spray aerosols (SSAs). Here, we conducted field experiments along a north-south transect of the Atlantic Ocean to study the enrichment of PFAAs in SSA. We show that in some cases PFAAs were enriched >100,000 times in the SSA relative to seawater concentrations. On the basis of the results of the field experiments, we estimate that the secondary emission of certain PFAAs from the global oceans via SSA emission is comparable to or greater than estimates for the other known global sources of PFAAs to the atmosphere from manufacturing emissions and precursor degradation.

## Teaser

The re-emission of certain PFAS from the oceans to air via sea spray is comparable to their emissions from other sources to air.

## INTRODUCTION

Per- and polyfluoroalkyl substances (PFASs) are a class of synthetic organic chemicals of global concern that are ubiquitous in the environment after decades of production and use in consumer and industrial applications (*1*). Perfluoroalkyl acids (PFAAs), the best-studied subgroup of PFAS, have been reported globally in precipitation, with the concentrations of certain long-chain PFAAs often above health advisories for drinking water (*2*). Temporal trends in the levels of PFAAs in the atmosphere at various remote monitoring stations vary depending on the origin of air masses, but despite their drastically reduced emissions (*3*, *4*), the levels of long-chain PFAAs in the atmosphere and precipitation have been relatively stable since measurements began (*5*). Given the concerns regarding the widespread and long-lasting atmospheric presence of PFAAs, understanding their sources, transport, and fate is an urgent research need.

PFAAs released into the environment are expected to be transported to the oceans and, due to their exceptional environmental persistence (*6*), it is believed that their sole sink is dilution into the deep oceans (*7*). Recent studies on the vertical distribution of PFAAs in the Atlantic Ocean have demonstrated that PFAA levels are highest in the upper layer of ocean water, with concentrations sharply decreasing as depth increases (*8*, *9*). Rising air bubbles scavenge PFAAs from seawater, causing them to accumulate at the bubbles’ air-water interface. Subsequently, these PFAAs are released into the air together with sea spray aerosol (SSA) by bubble bursting. Field studies demonstrate that PFAAs transported via SSA substantially contribute to their concentrations in coastal air (*10*, *11*) and potentially act as an important cause of elevated concentrations of PFAAs in soil, water, and vegetation in coastal regions (*12*, *13*). This remobilization of PFAAs from the ocean to the atmosphere and subsequent transport and deposition back to terrestrial environments results in the continuous and long-term cycling of PFAAs in the hydrosphere between terrestrial and marine environments, which may inhibit the decrease in global environmental levels of these very persistent substances.

In previous work, we attempted to estimate the contribution of SSA to the atmospheric loading of PFAAs (*14*). To do so, we measured the enrichment factors (EFs) of PFAAs in SSA, which are defined as the ratio between the concentrations of PFAAs in SSA and in water (normalized to the sodium concentration in the respective compartment), in a series of laboratory experiments (*14*) using a sea spray simulation chamber (*15*). On the basis of laboratory-derived EFs and the modeled global SSA emission, we estimated that the secondary emission of perfluorooctanoic acid (PFOA) and perfluorooctane sulfonic acid (PFOS) via remobilization in SSA from the global oceans is potentially greater than the two main known sources of PFAAs in the air, namely, direct emission from industrial sources and atmospheric transformation of other volatile PFAS, the so-called PFAA precursors (*14*). Although we demonstrated that the variation in PFAA concentrations in water has a minor influence on the enrichment process (*16*), the laboratory-derived EFs remain a potential source of uncertainty in the estimation. Our laboratory experiments were conducted using artificial seawater (*14*) or sodium chloride solution (*16*) at constant temperature and salinity; therefore, it is uncertain whether the laboratory-derived EFs are environmentally relevant.

Here, we performed a series of field experiments using a sea spray simulation chamber (fig. S1) deployed on a cruise along a transect from ~50°N to 50°S on the Atlantic Ocean (fig. S2). During the cruise, we continuously pumped fresh seawater into the chamber to generate SSA and used a cascade impactor to separate the nascent SSA into eight size fractions (0.015 to 15 μm). We aimed to investigate the enrichment of PFAAs in different SSA size fractions under environmentally relevant conditions. On the basis of these field-derived EFs, we have estimated the secondary emission of PFOA and PFOS from the global oceans.

## RESULTS

**Fig. 1.**
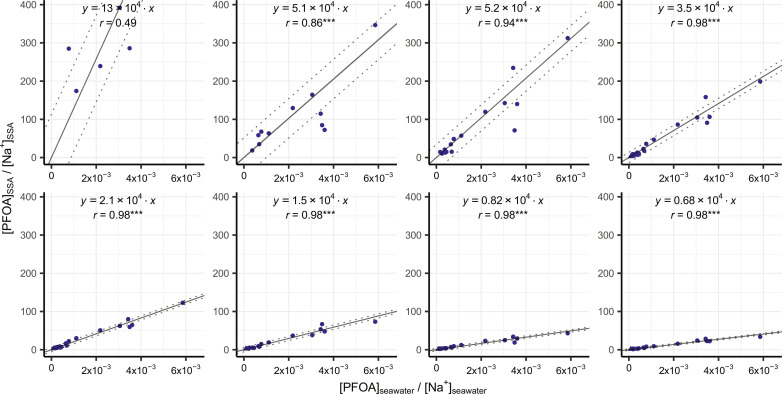
Linear relationship between PFOA concentration in SSA and in seawater. PFOA concentrations in both SSA and seawater are normalized to Na^+^ concentrations. MDL_water_ was used for concentrations in the chamber water below the detection limits. SSA samples below the MDL_SSA_ were excluded. The dashed lines indicate ±σ. The number of asterisks indicates *P < 0.05, **P < 0.01, and ***P < 0.001. Plots for other PFAAs can be found in fig. S3.

**Fig. 2.**
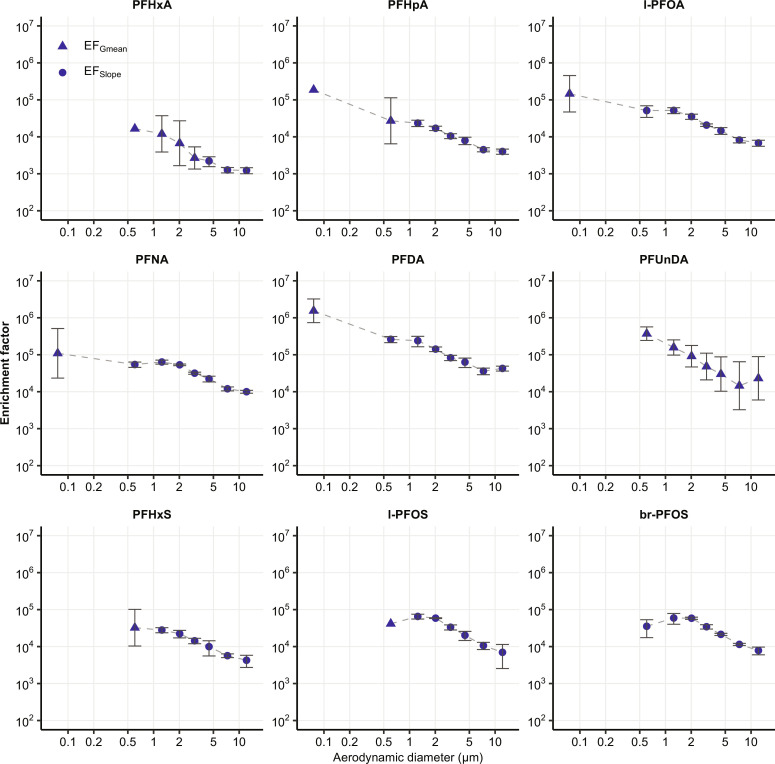
EFs of PFAAs derived from the field experiments. The circle markers (EF_Slope_) represent EFs estimated based on the slope of the total least squares linear regression. The triangular markers (EF_Gmean_) represent the geometric mean of the EFs calculated individually for each experiment. Error bars represent the 95% confidence interval (α = 0.05).

We analyzed SSA samples and the seawater inside the SSA chamber for both PFAAs and Na^+^. The results (Fig. 1 for PFOA and fig. S3 for all PFAAs) show significant linear relationships (*P* < 0.05) between PFAA concentrations in SSA particles (normalized to Na^+^ concentration, [*PFAA*]_SSA_/[*Na*^+^]_SSA_) and their concentrations in water across most size fractions ([*PFAA*]_seawater_/[*Na*^+^]_seawater_). Figure 2 shows the mean EFs for each individual PFAA in different size fractions (see table S1 for the details). We determined the mean EFs by calculating the slope of the total least squares linear regression between PFAA concentrations in water ([*PFAA*]_seawater_/[*Na*^+^]_seawater_) and in SSA ([*PFAA*]_SSA_/[*Na*^+^]_SSA_), but only if the correlation was significant (*P* < 0.05). In cases where the correlations were insignificant (*P* > 0.05), we used the geometric means of the EFs calculated for each experiment in the subsequent analysis.

Generally, the linear relationship is strong in the supermicrometer size fractions (Pearson’s *r* > 0.9) but weaker or even insignificant (*P* > 0.05) in the submicrometer size fractions. Particle size (*d*_p_) significantly influences the enrichment on supermicrometer SSA, as the EFs of individual PFAAs exhibit a strong correlation with *d*_p_ in a log-log linear relationship (Pearson’s *r* > 0.9, *P* < 0.01; table S2). The differences in the results for the supermicrometer and submicrometer size fractions are hypothesized to be due to the different formation mechanisms of these types of SSA (see Supplementary Text). It should also be noted that the PFAAs in the submicrometer size range were often close to the method detection limits (MDLs), which may be a cause of the weaker or insignificant correlation for some homologs and size fractions.

On the basis of (i) the mean EFs (table S3), (ii) concentrations of PFAAs obtained from our field experiments and from the literature (fig. S4 and table S4), and (iii) the global inorganic SSA emission and deposition fluxes (kg m^−2^ year^−1^) modeled by the Norwegian Earth System Model version 2 (NorESM2; fig. S5) (*17*–*19*), we were able to estimate the annual secondary emission of PFOA and PFOS remobilized from the oceans through SSA emission (expressed as t year^−1^, details in Materials and Methods), taking into account both linear and branched isomers. Hereafter, when we mention “emission/deposition,” we are referring to the secondary emission of PFOA or PFOS that occurs when they are remobilized through SSA emission and then subsequently deposited, unless specified otherwise. Table 1 summarizes the results of the estimation.

**Table 1.** Estimated emission and deposition of PFOA and PFOS via SSA. The spatial coverage of coastal provinces and different types of grid cells is shown in figs. S14 and S15, respectively.

	PFOA	PFOS	Inorganic sea salt
Estimated emission [mean (low–high)] (t year^−1^)	49 (29–91)	26 (15–37)	3.0 × 10^12^ kg year^−1^
Percentage from coastal provinces	33% (21–40%)	27% (20–32%)	7%
Percentage from other provinces	67% (79–60%)	73% (80–68%)	93%
Percentage associated with <1-μm SSA	6% (5–9%)	3% (2–4%)	2.5%
Estimated deposition [mean (low–high)] (t year^−1^)	51 (30–96)	27 (16–40)	
Percentage to inland cells	3.9% (3.3–4.1%)	2.6% (2.3–3.0%)	6%
Percentage to coastline cells	22% (16–26%)	17% (13–22%)	2%
Percentage by dry deposition	40%	40%	41%

We estimate that 49 (29 to 91) tons of PFOA and 26 (15 to 37) tons of PFOS are emitted annually from the global oceans through SSA (Table 1). These values can be compared with the estimates available for other atmospheric sources in the literature. Xie *et al.* (*20*) estimated that approximately 1 to 1.4 tons of PFOS were emitted into the air each year globally from industrial sources, and Wang *et al.* (*4*) estimated that <2.8 tons of PFOS were formed each year globally by degradation of precursor compounds between 2003 and 2015. Thus, the estimated emission of PFOS from the oceans to air through SSA exceeds the estimated contribution of other potential sources of PFOS to the atmosphere globally. For PFOA, the estimated emission from oceans to the air via SSA is comparable with the estimated 14 to 74 tons of PFOA derived from other atmospheric sources globally in 2012, including direct manufacturing emissions and precursor transformation (*3*).

**Fig. 3.**
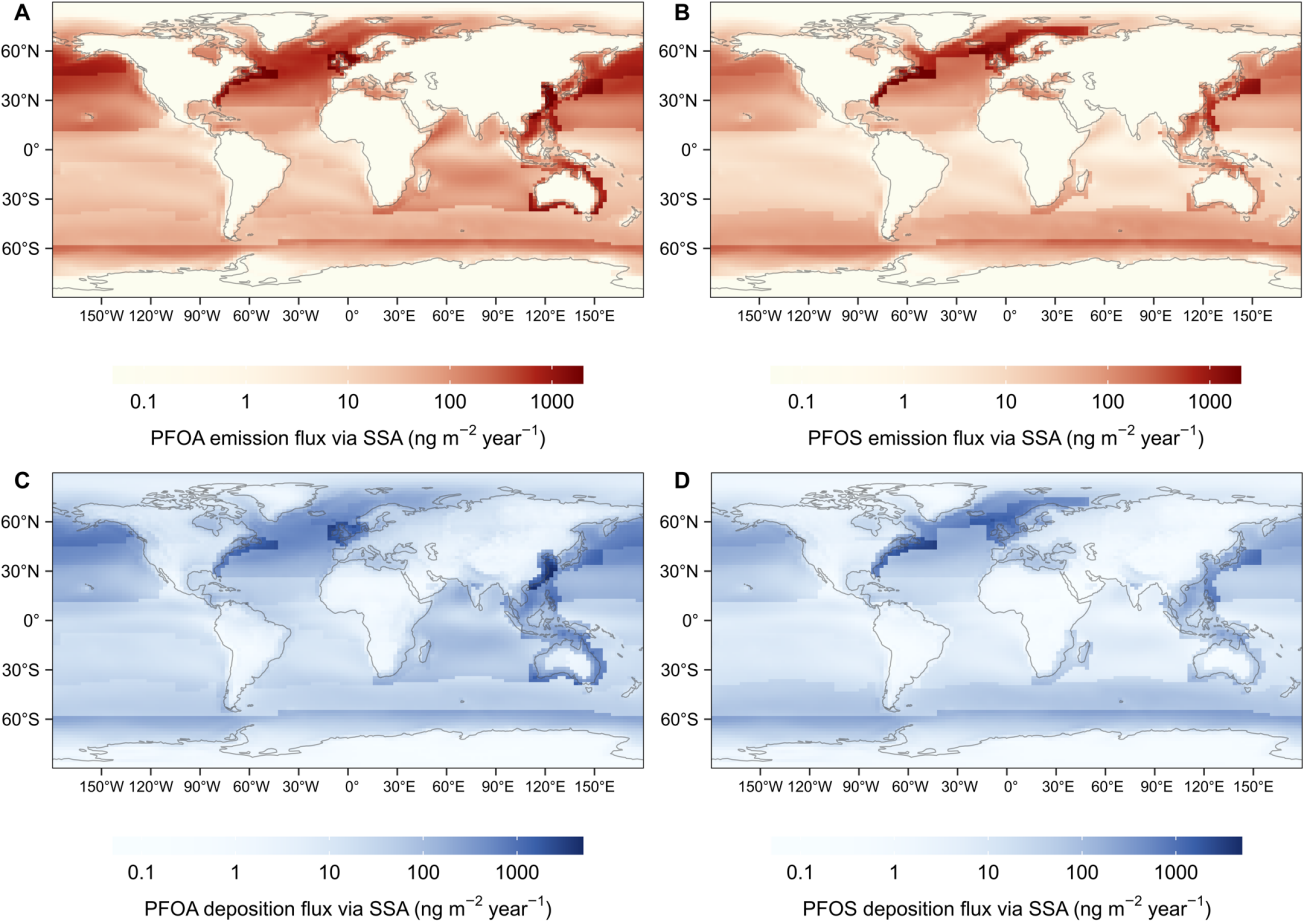
Spatial distribution of PFAA emission fluxes via SSA and deposition fluxes following the emission via SSA. (**A** and **C**) are for PFOA. (**B** and **D**) are for PFOS. The deposition fluxes in the figure refer to total deposition, including both wet and dry deposition.

Figure 3 shows the spatial distribution of the estimated flux of PFAA emitted via SSA and the total deposition flux (wet and dry deposition combined; ng m^−2^ year^−1^). The pattern of emission fluxes (Fig. 3, A and B) reflects the influences of both the geographical variation in the production of SSA (fig. S5) and the concentrations of PFAAs in seawater (fig. S4). While only a small fraction (7%) of SSA is produced in coastal regions, PFAA emitted from coastal waters accounts for 20 to 40% of the total PFAA emission on SSA (Table 1) due to the much higher concentrations of PFAAs in coastal seawater (fig. S4). Coastal regions with high PFAA concentrations in seawater, for example, the coast of China and the west coast of North America, can have very high emission fluxes (10^2^ to 10^3^ ng m^−2^ year^−1^). On the other hand, the emission flux from the open ocean can also be relatively high (10^2^ ng m^−2^ year^−1^) due to the greater SSA emission flux (fig. S5), such as in the North Atlantic.

We estimate that approximately 15 to 30% of the PFAA emitted on SSA from the global oceans is transported and deposited to land, with 13 to 26% deposited to coastline grid cells and 2 to 4% to inland grid cells (Table 1). The total deposition fluxes in land cells decrease from the coastline toward inland areas, as indicated in Fig. 3, C and D (fig. S6 shows this in detail). Most (70 to 85%) of the PFAA emitted on SSA is deposited back to the oceans. The wet deposition fluxes are generally greater than the dry deposition fluxes, and on average, wet deposition contributes 60% of the estimated global deposition of PFOA and PFOS via SSA.

## DISCUSSION

**Fig. 4.**
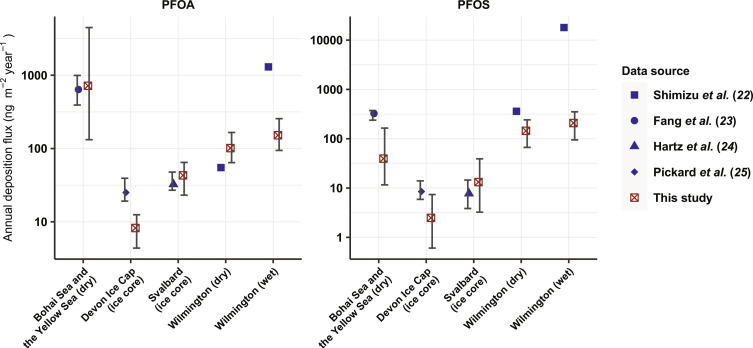
Comparison between PFAA deposition fluxes calculated based on environmental samples and deposition fluxes via SSA estimated in the present study. The blue markers, and the lower and the upper bounds of the error bars, indicate the median values, and the first and third quartiles of the deposition fluxes reported in field studies (*22*–*25*), respectively. The red markers, and the associated upper and lower bounds of the error bars, indicate the mean values, and the low and high emission scenarios, respectively, from this study. The blue and red markers indicate total deposition fluxes (including both wet and dry deposition) if not specified in brackets. The annual deposition fluxes by Fang *et al.* (*23*) are extrapolated from daily fluxes. Only deposition fluxes for years after 2005 by Hartz *et al.* (*24*) and Pickard *et al.* (*25*) are considered.

Our estimates suggest that the remobilization of PFOA and PFOS on SSA is an important contributor to atmospheric PFAAs, and that the contribution can be comparable to or even greater than other known sources. Thackray *et al.* (*21*) modeled global PFOA deposition fluxes following direct industrial emission and PFAA precursor transformation. In open ocean and coastal regions, we estimate that the total deposition flux of PFOA transported on SSA is generally between 10 and 1000 ng m^−2^ year^−1^ (Fig. 3C), which is of the same order of magnitude as the deposition fluxes modeled by Thackray *et al.* (*21*). This suggests that the remobilization of PFAAs on SSA may play a considerable role in contributing to PFOA levels in these regions, comparable in importance to the aforementioned sources. The deposition fluxes in our estimation are also comparable with the calculated PFAA dry deposition fluxes based on field measurements (*22*, *23*) and total deposition fluxes derived from ice core samples (*24*, *25*) as shown in Fig. 4, indicating that PFAA transported on SSA can potentially contribute substantially to deposition fluxes. The impact of the remobilization of PFAAs on SSA likely increases with the increasing intensity of other PFAA sources in coastal regions due to the elevated concentration in coastal seawater as a result of direct industrial emissions (*26*, *27*) and/or transformation of PFAA precursor compounds.

**Fig. 5.**
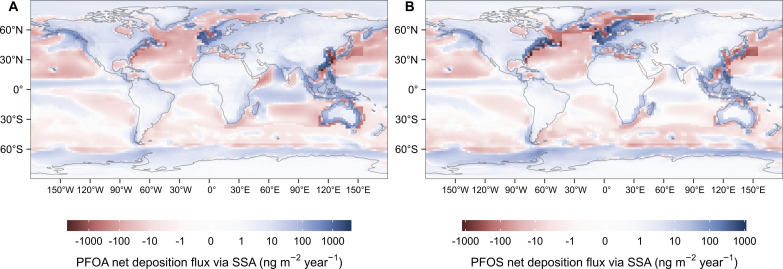
Difference between PFAA emission fluxes and deposition fluxes via SSA. Positive values indicate that the deposition fluxes are greater than the emission fluxes. (**A**) is for PFOA, and (**B**) is for PFOS.

Figure 5 shows the differences between the emission fluxes and deposition fluxes of PFAAs on SSA. The PFAA emission fluxes from the oceans are generally greater than the total deposition fluxes to the oceans, except in regions around the equator. This indicates that the open oceans, for example, the North Atlantic between 30°N and 60°N, could be important for contributing PFAAs to air. This is consistent with our previous air trajectory analysis of aerosol samples collected from Andøya (16.00°E, 69.27°N) and Birkenes (8.25°E, 58.38°N), Norway, which showed that the North Atlantic could contribute to the measured concentrations of PFOA at the sampling sites (*10*). The calculated atmospheric PFAAs/Na^+^ ratios based on the NorESM2 results at the two Norwegian coastal locations were at the higher end of the ratios in these aerosol samples, suggesting that the NorESM2 result may overestimate the impact of PFAAs transported on SSA at these two sites (see fig. S7 and Supplementary Text).

Here, we investigated the enrichment of PFAAs on SSA under natural seawater conditions. The EFs were less varied in the supermicrometer size range than in the submicrometer size rang as indicated by the stronger linearity between the PFAA concentrations in supermicrometer SSA and in seawater (Fig. 1 and fig. S3). This suggests that the enrichment of PFAAs on supermicrometer SSA is likely less sensitive to changes in PFAA concentrations and other factors along the cruise, such as seawater temperature and salinity. We observed correlations between the field EFs of certain PFAAs and physical properties of seawater, such as temperature and salinity, in specific size fractions (summarized in table S5), but drawing definitive conclusions based on the correlation patterns is challenging due to the simultaneous variation of multiple parameters. Nonetheless, the greater uncertainty in the submicrometer EFs (fig. S3 and table S1) probably had only a minor impact on the estimated PFAA emission through SSA, since the PFAA emission associated with submicrometer SSA contributed approximately 5 to 9% and 2 to 4% of the total PFOA and PFOS emission from the global oceans (Table 1), respectively, and up to 23% of the total deposition fluxes of PFOA and PFOS in some inland regions (fig. S8).

The EFs determined in our field experiments were found to be higher than the EFs calculated using PFAA and Na^+^ concentrations in ambient aerosol samples and nearby seawater samples reported in a previous study (*28*). This disparity can be attributed to the fact that fine particles in the atmosphere have a long residence time, and ambient SSA collected at the sampling site may originate from distant sea regions. Consequently, the EFs calculated from ambient aerosol samples and nearby seawater samples may differ from the EFs observed in the nascent SSA directly generated in the chamber.

Our field-derived EFs were higher than the EFs determined in laboratory experiments (fig. S9) using the same SSA simulation chamber filled with sodium chloride solution at constant salinity and water temperature (*16*). After the cruise, we carried out a series of additional laboratory experiments to investigate the potential factors influencing the enrichment process (see Supplementary Text). We found that the addition of natural organic matter (OM) to artificial seawater at a concentration of 1 mg liter^−1^ total organic carbon (TOC) resulted in a significant increase (*P* < 0.05) of up to five times in the EFs, compared to the experiment without the addition of OM. This increase depended on the length of the perfluoroalkyl chain and the SSA size fraction (fig. S10). These findings are consistent with similar results reported by Li *et al.* (*29*). The typical concentration of dissolved organic carbon in open ocean seawater is generally <1 mg liter^−1^ (*30*). Therefore, the presence of OM in natural seawater may explain the higher enrichment observed in field studies compared to previous laboratory experiments. Further, increasing the TOC concentration in the chamber water from 1 mg liter^−1^ to 2 mg liter^−1^ resulted in a slight reduction of EFs in <5-μm size fractions by 10 to 60% on average (fig. S10). This suggests that higher concentrations of OM in seawater may act to reduce the enrichment process. However, the mechanism behind the enhancement of EFs requires further research, since the organic composition in water can influence both the behavior of PFAAs at the air-water interface (*31*) and SSA production (*32*). It is important to note that the additional laboratory experiments used the Nordic humic acid standard from the International Humic Substances Society (IHSS), which has a different composition compared to the OM present in natural seawater. Nonetheless, these preliminary results indicate that the presence of OM in seawater could be a major factor contributing to the discrepancy between the field experiments and laboratory experiments without the addition of OM, and the enrichment of PFAAs on SSA may vary greatly from the more polluted coastal waters to the open ocean regions due to the greater difference in OM concentration and/or OM composition (*30*, *33*).

The variation in PFAA concentrations in the oceans and the scarcity of field measurements could be important sources of uncertainty when estimating the remobilization of PFAAs from the oceans. In our estimates, we partitioned the global ocean based on the Longhurst biogeochemical ocean provinces (*34*). There are 54 provinces in total, 22 of which are coastal provinces (fig. S11 and table S4). While field measurements of PFOA and PFOS in seawater are only available for 26 ocean provinces, the measurements are unevenly distributed (table S4 and fig. S12). There is an even greater paucity of data for the coastal provinces since only 8 of the 22 coastal provinces have available PFOA and PFOS measurements (table S4). In our estimates, we used the concentrations in neighboring open ocean provinces for the coastal provinces without any field measurements (see Materials and Methods for details). However, it is known that the concentrations vary by several orders of magnitude from populated coastal regions to the open oceans (*35*), which can lead to underestimation for these coastal regions. Additionally, our modeling is not optimized for SSA production in the surf zone, which is probably more related to wave energy dissipation than wind speed and may lead to a higher SSA emission flux (*36*). This implies that our estimation based on open ocean conditions was conservative. It is difficult to accurately assess how the variation in the concentration of PFAAs affects the patterns of emission fluxes and the impact of SSA-transported PFAAs on the local and global scale without good knowledge of the spatial distribution of PFAAs in oceans and SSA production in the surf zone. Therefore, we encourage the scientific community to increase monitoring efforts of PFAAs in the global oceans. Measurements in the North Pacific and Arabian Sea are especially interesting as these waters are potential hotspots with high PFOA emission fluxes (Fig. 3).

To summarize, we demonstrate that PFAAs are highly enriched in nascent SSA and estimate that they are remobilized from the oceans in substantial quantities to the atmosphere as a consequence. These findings have implications for human exposure to PFAAs, especially in coastal regions, and this merits further investigation. For example, our estimates include neither the contribution of shoreline wave breaking to the atmospheric burden of PFAAs nor the influence of the higher concentrations of PFAAs generally found in coastal regions. As such, our estimates on the deposition of PFAAs to coastal regions following their remobilization through SSA are likely to be conservative.

## MATERIALS AND METHODS

### Target compounds

In total, we investigated 10 PFAAs here, including 7 perfluoroalkyl carboxylic acids (PFCAs) and 3 perfluoroalkane sulfonic acids (PFSAs). The seven PFCAs are perfluorohexanoic acid (PFHxA), perfluoroheptanoic acid (PFHpA), PFOA, perfluorononanoic acid (PFNA), perfluorodecanoic acid (PFDA), perfluoroundecanoic acid (PFUnDA), and perfluorododecanoic acid (PFDoDA). The three PFSAs are perfluorobutane sulfonic acid (PFBS), perfluorohexane sulfonic acid (PFHxS), and branched and linear PFOS (table S6).

### Field experiments

We conducted the field experiments on board the *Royal Research Ship (RSS) Discovery* during the 29th Atlantic Meridional Transect (AMT29). The cruise departed from Southampton in the UK on 13 October 2019 and arrived at Punta Arenas in Chile on 26 November 2019, covering a meridional transect from approximately 50°N to 50°S (fig. S2).

We used a sea spray simulation chamber (fig. S1) developed by Salter *et al.* (*15*) to generate SSA. The chamber is 47 cm in diameter and 100 cm in height and is made of stainless steel. All surfaces below the water level on the inside are coated with polytetrafluoroethylene (PTFE). During the cruise, seawater from the ship’s underway seawater supply (~5 m below the sea surface) was continuously pumped through a stainless steel nozzle (inner diameter, 4.3 mm) at the center of the chamber’s lid by a peristaltic pump (Watson-Marlow, 620S) at 3.2 liters min^−1^ to create a plunging jet. The jet entrained air into the bulk water, and, when the air bubbles burst at the water surface, aerosols were released to the headspace. To keep the water level constant, another peristaltic pump (Watson-Marlow, 620S) continuously pumped water out of the chamber from the bottom at a slightly lower flow rate (~3.17 liters min^−1^) and a tap on the side of the chamber located 60 cm above the bottom was kept open to let excess water flow out of the chamber.

During each experiment, SSA in the chamber’s headspace was sampled using a 14-stage cascade impactor (DLPI+, Dekati) at a flow rate of 9.6 liters min^−1^ for ~30 hours. SSA of different aerodynamic diameters was separated onto polycarbonate membranes (Nuclepore Track-Etch Membrane, Whatman) on the impactor stages. The cutoff sizes (*d*_50_) of the stages ranged from 0.015 μm to 9.91 μm (table S7). The sampling line was heated to keep the relative humidity at the impactor inlet below 40% so that SSAs were completely dried before entering the impactor. Particle-free sweep air (~20 liters min^−1^) was introduced to the chamber to prevent possible contamination from indoor air. The chamber water was sampled continuously in triplicate by pumping through three Oasis weak-anion exchange (WAX) solid-phase extraction (SPE) cartridges (6 cm^3^, 500 mg, 30 mm) using a multichannel peristaltic pump via a tap on the side of the chamber. The water volume that passed through each SPE cartridge was ~10 liters. The salinity and temperature of the water in the chamber were continuously logged, and 10 ml of chamber water was collected at 0, 15, and 30 hours of each experiment for ion analysis. The water temperatures measured in the chamber were <1.1°C lower than the values recorded by the ship’s underway monitoring system. Despite technical issues with the salinity sensor in the chamber, it can be assumed that the salinity remained consistent between the chamber and the underway system, given that salinity is a conservative property. Consequently, only the data from the ship’s underway system were used in the subsequent analysis. In total, 19 experiments were conducted. Detailed information about the experiments, such as the start and end location, sampling duration, and water volume, is provided in table S8.

### Extraction and instrumental analysis

After each experiment, the polycarbonate membranes from the impactor stage 1 to stage 6 were pooled and stages 7 and 8 were pooled, so the 14 impactor stages resulted in eight size fractions (table S7). The membranes were placed in 10-ml polypropylene (PP) tubes and stored at −18°C. The extraction was performed after the cruise at Stockholm University. The membranes were first sonicated in 10 ml of MilliQ water for 30 min. After taking a 0.5-ml subsample for ion analysis, the remaining aliquots were spiked with a mixture of mass-labeled internal standards (IS) and concentrated on WAX SPE cartridges based on a previously published method (*37*). Briefly, the cartridges were prewashed with 4.5 ml of 0.3% NH_4_OH in methanol before applying the samples. Then, the cartridges were washed with 5 ml of 20% MeOH in MilliQ, followed by 2 ml of 0.3% NH_4_OH in MilliQ, and finally eluted with 6 ml of 0.3% NH_4_OH in methanol. The membranes were sonicated again with 3 ml of methanol for 15 min, and the extract was combined with the SPE eluent.

For the chamber water samples, the prewash and activation (4.5 ml of 0.1 mM formic acid in MilliQ water) of the cartridges were performed before the cruise. The cartridges were wrapped in aluminum foil, sealed in zip bags, and stored at 4°C before use. The extraction of the water samples was conducted on board during each experiment as described previously. Then, the used cartridges were spiked with a mixture of mass-labeled IS and stored at −18°C. The wash and elution of the cartridges was conducted after the cruise together with the SSA samples. The eluent of the SSA and water samples was evaporated to dryness and reconstituted in 50% methanol and 50% 4 mM ammonium acetate in MilliQ water to a final volume of 300 μl. Recovery standards (RS) were added before instrumental analysis.

The target compounds were analyzed on an Acquity ultra-performance liquid chromatography system coupled to a Xevo TQ-S tandem mass spectrometer (UPLC/MS/MS; Waters Corp.) based on a previously published method (*38*). Briefly, 50 μl of the final extract was injected on an Ascentis Express F5 PFP Column (2.7 μm, 10 cm × 2.1 mm, Sigma-Aldrich) equipped with an Ascentis Express F5 PFP guard column (2.7 μm, 5.0 mm × 2.1 mm), both maintained at 30°C. A “PFC isolator column” obtained from Waters “PFC kit” was placed before the injector to delay background contamination originating from the UPLC instrument and mobile phase. The mobile phase consisted of (A) 2 mM ammonium formate and 2 mM formic acid in MilliQ water and (B) MeOH. The flow rate of the mobile phase was 0.25 ml/min, and the mass spectrometer was operated in negative electrospray ionization mode. Gradient conditions were as follows: 90% B for 1 min, 40% B by 3 min, 12% B by 14 min, 0% B by 14.5 min, then 90% B and equilibrate for 6.5 min. Sodium concentrations in the SSA and chamber water samples were determined by chemically suppressed ion chromatography (IC; Dionex ICS-2000) using CG16/CS16 columns.

Details on the determination of MDLs and method quantification limits (MQLs) are described in Supplementary Text. The MDLs, MQLs, and PFAA detection frequencies and concentrations in water and in SSA are presented in tables S9 to S11.

### EF calculation

The EF is defined according to Eq. 1:

$$\text{EF}=\frac{{\left[\text{PFAA}\right]}_{\text{SSA}}/{\left[{\text{Na}}^{+}\right]}_{\text{SSA}}}{{\left[\text{PFAA}\right]}_{\text{seawater}}/{\left[{\text{Na}}^{+}\right]}_{\text{seawater}}}$$ (1)

where [*PFAA*]_SSA_ and [*PFAA*]_seawater_ are the concentrations of PFAAs in SSA and seawater, respectively, and [*Na*^+^]_SSA_ and [*Na*^+^]_seawater_ are the concentrations of the SSA tracer ion, Na^+^, in SSA and seawater, respectively.

In each individual experiment, we calculated the EFs of PFAAs using Eq. 1. If there was a significant correlation between the PFAA concentrations in SSA and the concentrations in water, we estimated the mean EFs using the slope of the total least squares linear regression (*EF*_slope_) as shown in Eq. 2:

$$\frac{{\left[\text{PFAA}\right]}_{\text{SSA}}}{{\left[{\text{Na}}^{+}\right]}_{\text{SSA}}}=\text{EF}\times \frac{{\left[\text{PFAA}\right]}_{\text{seawater}}}{{\left[{\text{Na}}^{+}\right]}_{\text{seawater}}}$$ (2)

In cases where the correlation was not significant, the geometric means of the EFs were used (*EF*_Gmean_). EFs were only calculated for PFAAs with detection frequencies in the chamber water >30%. Values that were between the MDL and MQL were used in EF calculations as they were quantified in the analytical method. MDL_water_ was used for concentrations in the chamber water below the detection limits. The calculated EFs for each size fraction are presented in Fig. 2 and table S1.

### Estimation of PFAA emission from the oceans via SSA

We estimated PFAA remobilized from the global oceans via SSA (*Flux*,_PFAA_, t year^−1^) by rearranging Eq. 1:

$${\text{Flux}}_{\text{PFAA}}={\text{EF}}_{\text{PFAA}}\times {\text{Flux}}_{{\text{Na}}^{+}}\times \frac{{\left[\text{PFAA}\right]}_{\text{seawater}}}{{\left[{\text{Na}}^{+}\right]}_{\text{seawater}}}$$ (3)

where [*Na*^+^]_seawater_ is the mean Na^+^ concentration in surface seawater (10.8 g liter^−1^) and *Flux*_Na^+^_ (10^12^ kg year^−1^) is the annual emission of Na^+^ via SSA. We produced a mean and low and high emission scenario based on the confidence interval of the EFs and the variation of PFAA concentrations in the oceans, which are explained below.

The mean annual inorganic SSA emission between the years 2008–2014 modeled by the Norwegian Earth System Model version 2 (NorESM2) (*39*) was used in the estimation. The composition of inorganic SSA was assumed to be the same as inorganic sea salt, with Na^+^ accounting for 30.7% of the dry weight on average. The NorESM2 simulations for inorganic SSA are run with CMIP6 historical emissions for the years 2008–2014 (2007 was used as a spin-up year). To be comparable to observations, the meteorology was nudged to ERA-Interim data using a relaxation time of 6 hours (*40*). NorESM2 (*18*, *41*) was run with CAM6-Nor (*17*, *18*) coupled to the Community Land Model version 5 (CLM5) (*42*) in BGC (biogeochemistry) mode and prognostic crops. The resolution is 1.9° (latitude) × 2.5° (longitude) with 32 height levels from the surface up to ∼2.2 hPa in hybrid sigma coordinates. We use prescribed sea surface temperature (SST) and sea ice concentrations at 1.9 × 2.5° resolution (*43*). See Blichner *et al.* (*19*) for further details about the simulations [we use the default model simulations from Blichner *et al.* (*19*) in this study]. The output from NorESM2 includes monthly average of SSA emission flux (kg m^−2^ s^−1^), column burden (kg m^−2^), and wet and dry deposition flux (kg m^−2^ s^−1^). The modeled inorganic SSA emission by NorESM2 is around the first quartile of the inorganic SSA emissions computed by 12 chemical transport and general circulation models (*44*).

The empirical inorganic SSA source function used in NorESM2 was developed using the same sea spray simulation chamber as in the present study and consists of three log-normal modes: 0.095 μm, 0.6 μm, and 1.5 μm. (*45*). For each mode, the percentages of the mass in the eight size fractions were determined based on the area under the mass-size distribution curve of the estimated SSA emission flux (fig. S13). We then calculated the EFs for each mode according to the mass-size distribution. We used the mean EF (*EF*_slope_ or *EF*_Gmean_) and the lower and upper 95% confidence interval of the EF in the corresponding size range in the mean and low and high emission scenario, respectively (table S3). Since there was no significant difference in the EFs between branched PFOS and linear PFOS (as shown in table S1), the EFs used in the estimation of PFOS considered both the linear and branched isomers collectively.

PFAA concentrations in seawater varied greatly from populated coastal regions to the open ocean (*35*). To account for this geographical variability, we partitioned the global oceans according to Longhurst’s biogeochemical ocean provinces (*34*) and determined the PFAA concentrations in each province (figs. S4 and S11 and table S4) based on the field measurements in this study as well as available data collected from the literature published after 2015 (table S12).

The Longhurst system is a widely accepted classification of the pelagic ocean (*46*), which is mainly based on the spatial variability of physical and biochemical properties, such as seawater temperature, salinity, mixing state, chlorophyll concentration, and primary production. The oceans are categorized into four primary biomes (polar, westerlies, trade winds, and coastal) and then further partitioned into 54 biogeochemical ocean provinces, which are the regional expression of the biomes (*34*). To determine the PFAA concentrations for each Longhurst province, first, we overlaid a shapefile of the Longhurst provinces (http://marineregions.org/mrgid/22538) over the raster output from NorESM2 to determine the coverage of the provinces on the gridded map (fig. S11). We then identified the grid cells with available PFAA measurements based on the AMT29 cruise track as well as the sampling locations (latitudes and longitudes) extracted from literature (fig. S12 and table S12). We only considered data published after 2015 to avoid the influence of a potential time trend of PFAA concentrations in different regions of the global oceans (*35*). We used the reported MDLs in each study for the field data that were below the detection limit. If multiple field measurements were located within the same grid cell, we used the median value of these measurements as the PFAA concentration in that grid cell. For provinces that have PFAA concentration data in less than three grid cells, they were merged with an adjacent province with more data points. For the provinces that have PFAA concentration data in more than three grid cells (including merged provinces), we used the median, first quartile, and third quartile of the concentrations in the mean and low and high emission scenario, respectively. For provinces with no PFAA data, we determined the concentrations based on data in adjacent noncoastal provinces or use the Atlantic provinces at similar latitudes as references if the neighboring provinces also have no data (which is common for the Pacific provinces). Details on the name and description of the provinces as well as the concentrations used in mean and low and high emission scenarios are provided in table S4.

We also estimated the global deposition fluxes of PFOA and PFOS via SSA using Eq. 3 by replacing the SSA emission fluxes for SSA deposition fluxes. Given that (i) most SSA are produced on the open oceans (Table 1) and that (ii) most PFAAs on SSA are associated with large particles (*16*), which have relatively shorter atmospheric residence time (*36*), we assume that the relative contribution of SSA that originated from the open ocean likely increases with increasing distance from the coastline toward inland. To calculate the deposition fluxes on land, we extended the coastal provinces horizontally toward inland by two grid cells and use the PFAA concentrations of the coastal provinces for these grid cells. For the inland grid cells, we used the PFAA concentration in the closest noncoastal Longhurst province. All other parameters were the same. It should be noted that we directly applied the spatial pattern of PFAA concentrations in seawater (fig. S4 and table S4) to the raster output of NorESM2 using Eqs. 2 and 3, so it is not possible to track the origin of the deposited SSA. This could be a reason that total deposition estimates are slightly higher than emission estimates (Table 1).

To create the land mask and coastline mask, we used the land shapefile and coastline shapefile obtained from Natural Earth, which were at a scale of 1:110 m (available at: https://www.naturalearthdata.com/downloads/110m-physical-vectors/). By intersecting the raster map of the deposition flux with these shapefiles, we categorized grid cells that intersected with the land shapefile as “land cells,” and grid cells that intersected with the coastline shape file as “coast cells.” We categorized the land cells that do not intersect with the coastline shapefile as “inland cells.” Thus, “land cells” encompasses both “inland cells” and “coast cells.” Figure S14 shows the coverage of coastal and noncoastal Longhurst provinces, and fig. S15 shows the coverage of the different types of grid cells.

## Supplementary Materials

### This PDF file includes:

Supplementary Text Figs. S1 to S16 Tables S1 to S13 Legends for data S1 and S2 References
