# Supplementary material for: Constraining global transport of perfluoroalkyl acids on sea spray aerosol using field measurements
Source: Sci Adv. 2024 Apr 5;10(14):eadl1026. doi: 10.1126/sciadv.adl1026 (PMC10997204; doi:10.1126/sciadv.adl1026)
Supplement: Supplementary file 1 — Supplementary Text Figs. S1 to S16 Tables S1 to S13 Legends for data S1 and S2 References [file sciadv.adl1026_sm.pdf]

Science Advances
AAAS

Supplementary Materials for

Constraining global transport of perfluoroalkyl acids on sea spray aerosol using field measurements

Bo Sha *et al.*

Corresponding author: Bo Sha, bo.sha@aces.su.se

Sci. Adv. **10**, eadl1026 (2024)
DOI: 10.1126/sciadv.adl1026

The PDF file includes:

Supplementary Text
Figs. S1 to S16
Tables S1 to S13
Legends for data S1 and S2
References

Other Supplementary Material for this manuscript includes the following:

Data S1 and S2

Supplementary Text

QA/QC

As mentioned in the Materials & Methods section in the main text, the interior of the SSA chamber is coated with PTFE, which could potentially introduce contamination. To assess the impact of the PTFE coating on PFAA concentrations in the bulk water, we compared the l-PFOA concentration in the chamber water with the CTD samples collected at 2 – 5 m depth from 28 locations during the same cruise (8). The results demonstrate a strong agreement between the l-PFOA concentration in the chamber water and the surface CTD samples, particularly in the Southern Atlantic where PFOA levels are very low (fig. S16). Furthermore, considering that the two peristaltic pumps were operated at a flow rate of ~3.2 L min^{-1}, the turnover time for the 100 L of seawater in the chamber was approximately half an hour. This ensured a constant refreshment of the chamber with a large volume of fresh seawater throughout the experiments. As a result, any potential influence from the PTFE coating on the PFAA concentration is expected to be minimal.

The handling of the SSA samples on board was carried out in a glove box to prevent any contamination from indoor particles. Field blanks for SSA (n = 5 × 8 size fractions) were produced every four experiments by connecting the impactor loaded with polycarbonate membranes to the SSA chamber for 1 minute without sampling. Field blanks for the chamber water (n = 12) were produced by circulating ~5 mL MilliQ water through a WAX SPE cartridge during each experiment. PFOA was consistently detected in the field blanks for the chamber water samples at a level of 91±3 pg per SPE cartridge. For the other PFCAs, the concentrations in the field blanks were much lower at ~10 pg per SPE cartridge. The PFSA concentrations in the chamber water field blanks were generally <5 pg per SPE cartridge. For linear PFOA, the blank levels ranged from 2 to 42% of the values in the water samples before subtracting the blanks. In the case of branched and linear PFOS, the percentage ranged from 25 to 90%.

All results were blank corrected by subtracting the average concentration in the field blanks. The method detection limits (MDLs) for each of the target compounds were determined as 3×standard deviation (SD) of the blank levels if the target analytes were detected in the field blanks, and as 3 times the signal-to-noise levels (S/N) if the analytes were not detected in the field blanks. Method quantification limits (MQLs) were determined as 10×SD or 10×S/N, depending on whether the target analytes were detected in the field blanks. The water volume passed through each SPE cartridge during the field experiments varied between 8 – 13 L. In order to better represent the potential background contamination due to the materials and chemicals used in the extraction process, the field blanks, MDLs and MQLs were presented in "pg PFAA per SPE cartridge" instead of "pg L^{-1}" as shown in table S9. Combining the MDLs and MQLs in table S9 and the water volumes in table S8, the MDLs, MQLs or field blank levels of PFAAs in pg L^{-1} can be calculated for each experiment. For sodium analysis, all blanks were below the instrumental detection limit, so the MDL and MQL for Na^+ were derived from 3×S/N and 10×S/N, respectively.

Verification of the spatial distribution of PFOA and PFOS emission on SSA

Verification of the estimation requires measurements of both PFAAs and Na^+ in the same aerosol sample. As it is almost impossible to separate the contribution of different sources to atmospheric PFAAs, instead of directly comparing PFAA concentrations in air, the ratio between PFAA and Na^+ concentrations was used. In our previous study (*10*), aerosol samples were collected at two Norwegian coastal sites, Andøya (16.00°E, 69.27°N) and Birkenes (8.25°E, 58.38°N), between 2018 – 2020. The PFOA-to-Na^+ and PFOS-to-Na^+ ratios were calculated using Eq.2 based on the estimated SSA column burden in the grid cells with approximately same locations as Andøya and Birkenes from the NorESM model. The mean and the upper and lower 95% confidence interval (α=0.05) of the EFs of the three modes were used in the calculation. The calculated ratios were compared with the ratios in the aerosol samples as shown in fig. S7. Both transport through SSA and atmospheric transformation of PFAA precursor compounds can contribute to the concentration of PFAAs measured in the air samples from the two locations. Therefore, the calculated PFAA/Na^+ concentration ratios based on the NorESM result

was expected to be lower than the ratios in the air samples, or close to the ratios in the samples if transport through SSA was the dominant source of PFAAs. The 1st and 3rd quartile of the ratios in the air samples were used as criteria here: if the calculated ratios were greater than the 3rd quartile of the ratios in the samples, the remobilization/deposition of PFAAs through SSA may be overestimated; if the calculated ratios were lower than the 1st quartile of the ratios in the samples, the remobilization /deposition of PFAAs through SSA may be underestimated. The ratios in the mean emission scenario are comparable or even higher than the ratios in the aerosol samples, which suggest that the NorESM2 result may overestimate the impact of PFAAs transported on SSA. This is likely because the SSA collected at the two Norwegian coastal sites originated from both the coast and the open ocean, while in our estimation we assumed all SSA at the two sites was originated from the coast, where the concentrations of PFAAs in seawater are higher than the open ocean.

The correlation between PFAA concentration in SSA and in seawater in sub- and supermicron size fractions

In the present study we investigated the enrichment of PFAAs on SSA under natural seawater conditions and we observed linear relationships between the PFAA concentrations in SSA and in seawater (Fig.1 and fig. S3). We found that the strength of the linearity varies with the SSA particle size. Particle size (d_p) significantly influences the enrichment on supermicrometer SSA, as the EFs of individual PFAAs exhibit a strong correlation with d_p in a log-log linear relationship (Pearson's r>0.9, p<0.01, table S2). Supermicrometer SSA primarily consists of jet droplets, which are formed when bubbles collapse and disintegrate, releasing a small jet of water from the base of the bubble cavity (*47*). PFAAs within these jet droplets can be divided into two parts: the surface portion, which is scavenged by the air-water interface of the parent bubble, and the interior portion, which originates from the water in the surface microlayer (*48*). Thus, the portion of PFAA-enriched surface in relation to water, with lower PFAA concentration, decreases with increasing particle size.

For the submicrometer SSA, the linear correlations between PFAA concentrations in SSA and in water are weaker or statistically insignificant (p>0.05). Submicrometer SSA is mainly composed of film droplets that are produced after rupture of the bubble film cap (*6*). Seawater temperature, salinity, and the presence of organic matter can affect the production of film droplets (e.g. by influencing the size of air bubbles, the thickness of the film cap, etc.) as well as the interfacial behavior of PFAAs (*15, 31, 47*), and thus may affect the enrichment of PFAAs on film droplets. It should be noted that the PFAAs in the submicrometer size range were often close to the method detection limits (MDLs), which may be due to the weaker or insignificant correlation for some homologues and size fractions.

Laboratory experiments exploring the impact of organic matter on PFAA enrichment

Four groups of complementary experiments were carried out (table S13) using the SSA simulator at different conditions:

1. Exp-NaCl group: sodium chloride solution without organic matter (OM)
2. Exp-SS group: artificial seawater without OM
3. Exp-NOM1 group: artificial seawater with OM concentration equivalent to 1 ppm total organic carbon (TOC)
4. Exp-NOM2 group: Artificial seawater with OM concentration equivalent to 2 ppm TOC.

Each group consisted of three (triplicate) experiments at similar conditions. The chamber was filled with approximately 100 L of Milli-Q water (resistivity ~18 MΩ cm and total organic carbon (TOC) <3 μg L^{-1}).

In the Exp-NaCl group, the salinity of the water in the chamber was adjusted to ~37 psu using sodium chloride. In the Exp-SS, Exp-NOM1 and Exp-NOM2 groups, the salinity was adjusted to ~37 psu using artificial sea salt prepared following the method described by Kester et al. (*49*). In Exp-NOM1 experiments, 0.195 g of International Humic Substances Society (IHSS) Nordic humic acid standard was further added to the artificial seawater in the chamber, resulting in an aqueous TOC concentration of ~1 ppm. In the Exp-NOM2 experiments, 0.390 g of the IHSS humic acid standard was further added to the artificial seawater to achieve a TOC level of ~2 ppm. Before each group of experiments, the chamber water was fortified with a mixture of PFAAs to a concentration of ~3 ng L^{-1} for individual PFAAs. All experiments lasted 24 hours and the temperature of the chamber water was maintained at 15°C. Freshly generated SSA were directed through a heated sampling line into a 14-stages cascade impactor (Dekati DLPI+) at 9.6 L min^{-1}. Inside the cascade impactor, SSA particles were separated into 14 size fractions with cut-off size from 0.0157 µm – 9.91 µm. The 14 size fractions of the SSA samples were treated separately without pooling. The water in the chamber was sampled (500 mL) both before and after each experiment.

The SSA samples and chamber water samples were extracted and analyzed as described in the Materials & Methods section in the main text. The EFs were calculated according to Eq.1. The influence of organic matter and ion composition on the EFs was examined by testing whether the ratios between the EFs in two different experiment groups were significantly different from 1 (*t*-test, $\alpha = 0.05$). The ratios, R_{ij}, were calculated as EF_i/EF_j, where i and j denote triplicate experiment 1, 2 or 3 in experiment group A and B, respectively.

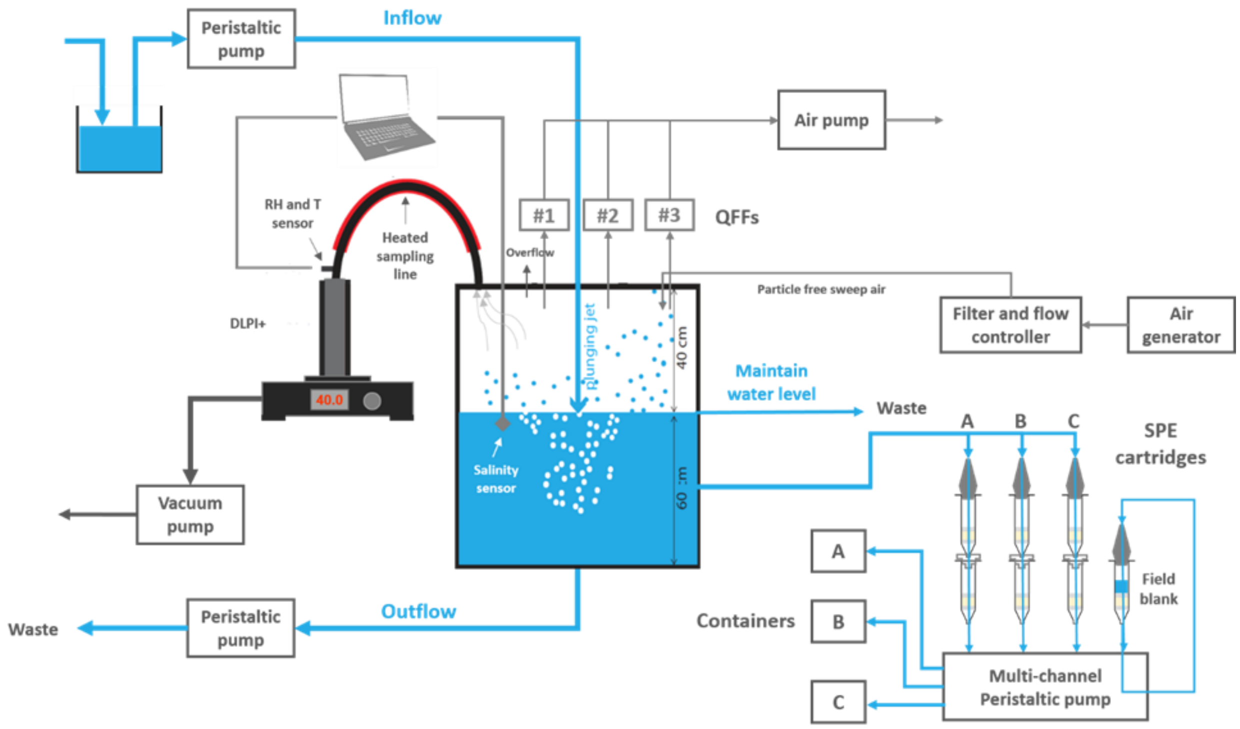

fig. S1.

Schematic of the sea spray chamber used to generate nascent SSA during the AMT29 cruise.

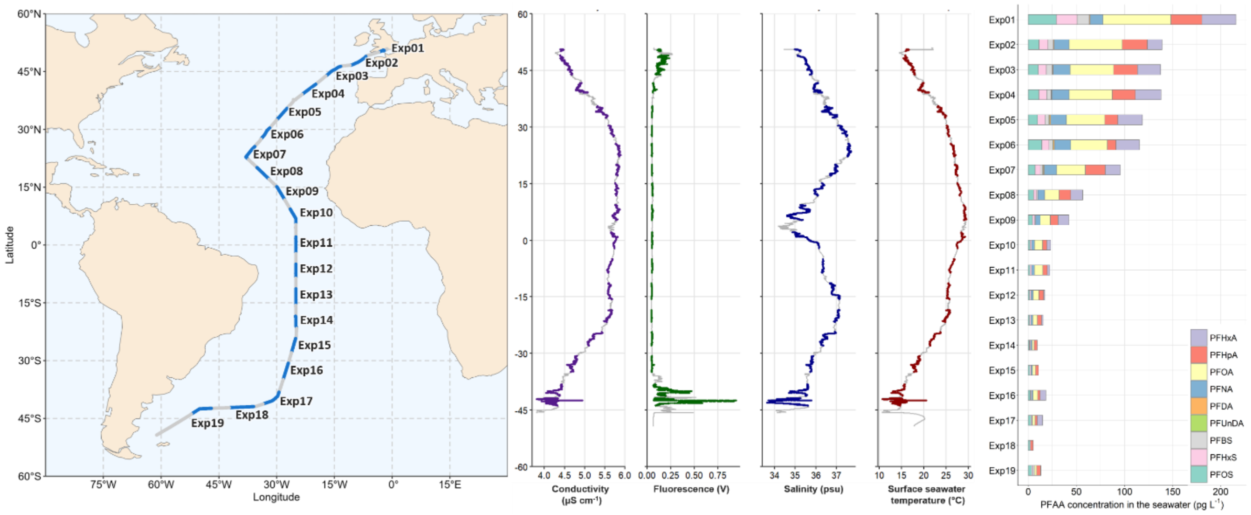

fig. S2.

Approximate locations of the chamber experiments during the cruise, physical properties of the seawater and concentrations of PFAAs measured in the chamber water. Salinity, conductivity and water temperature were recorded by the ship's underway monitoring system Abbreviations of the PFAAs can be found in table S8 in the Supplementary Material.

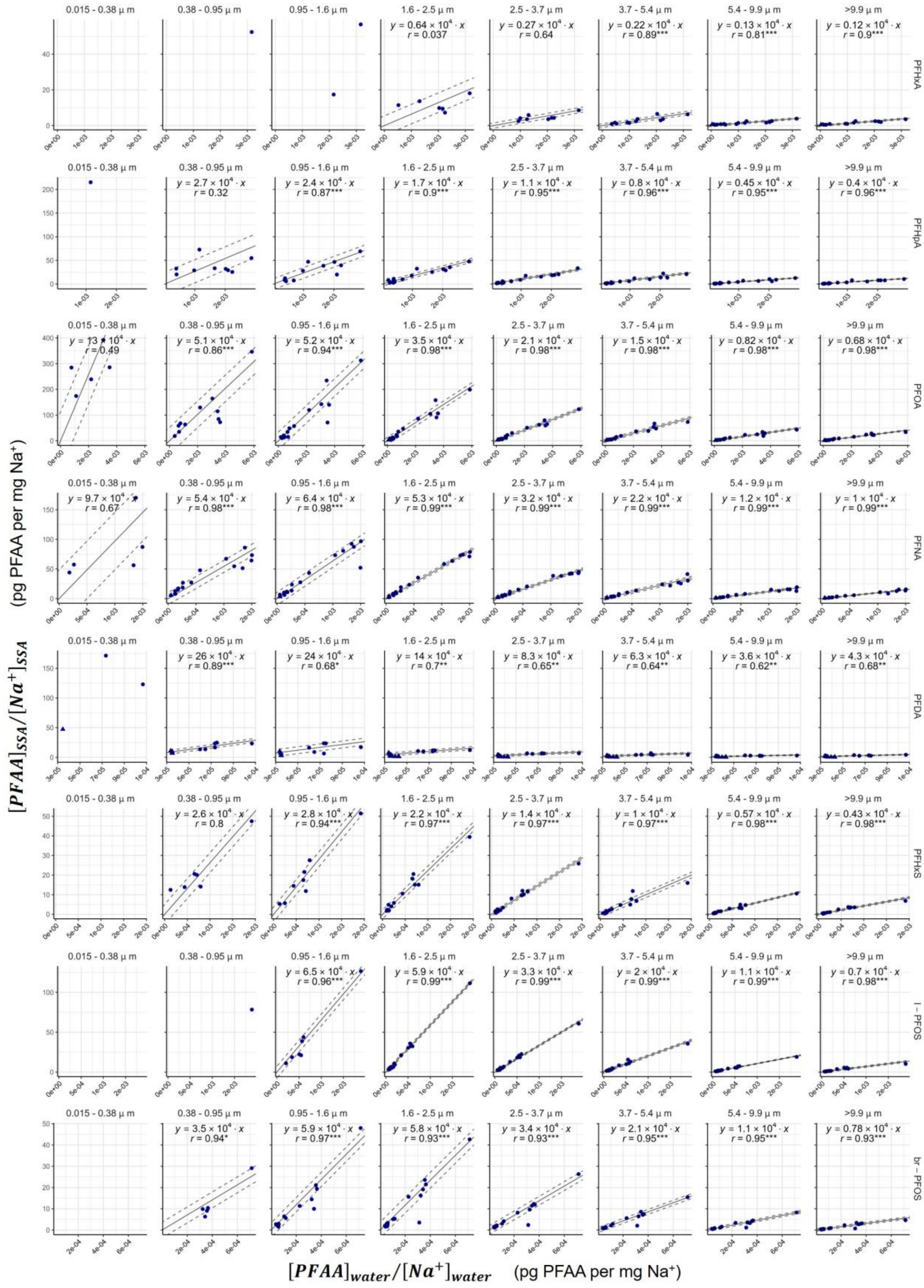

fig. S3.

Scatter plots of concentrations of PFAAs in the nascent SSA versus their concentrations in the corresponding chamber water samples in the eight size fractions. SSA sizes increase from left to right. Both PFAA concentrations in SSA and in chamber water are normalized to

Na^+ concentrations. Only PFAAs with detection frequencies in the chamber water >30% were included. MDL_{water} was used for concentrations in the chamber water below the detection limits. SSA samples below the MDL_{SSA} were excluded. The dashed lines indicate ±σ. The number of asterisks indicates $p<0.05$ (*), $p<0.01$ (**) and $p<0.001$ (***).

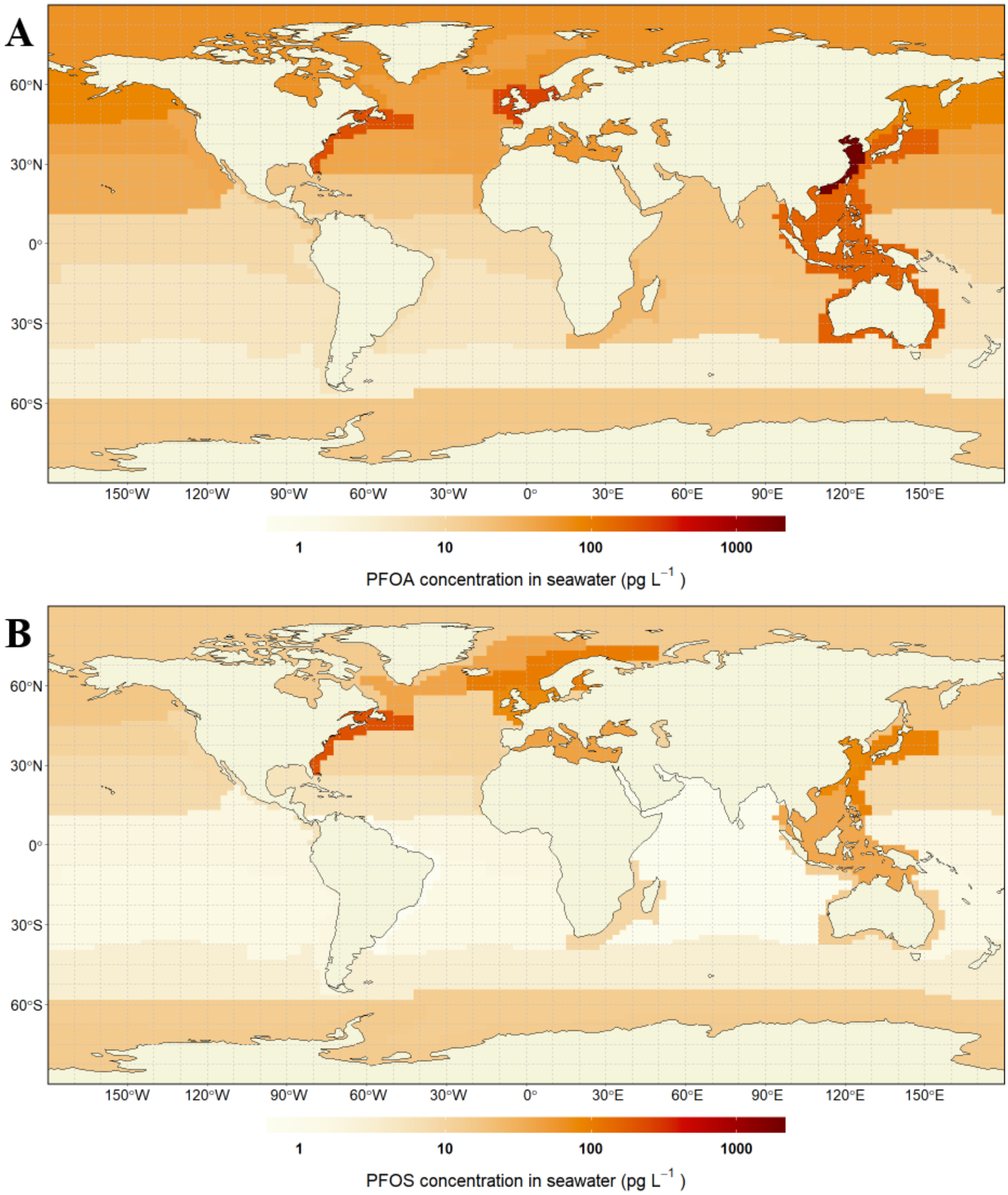

fig. S4.

Concentration of PFAAs in seawater for the Longhurst ocean provinces used in the median emission scenario. Panel A is for PFOA and Panel B for PFOS. For the Longhurst ocean provinces please refer to fig. S11 and table S4.

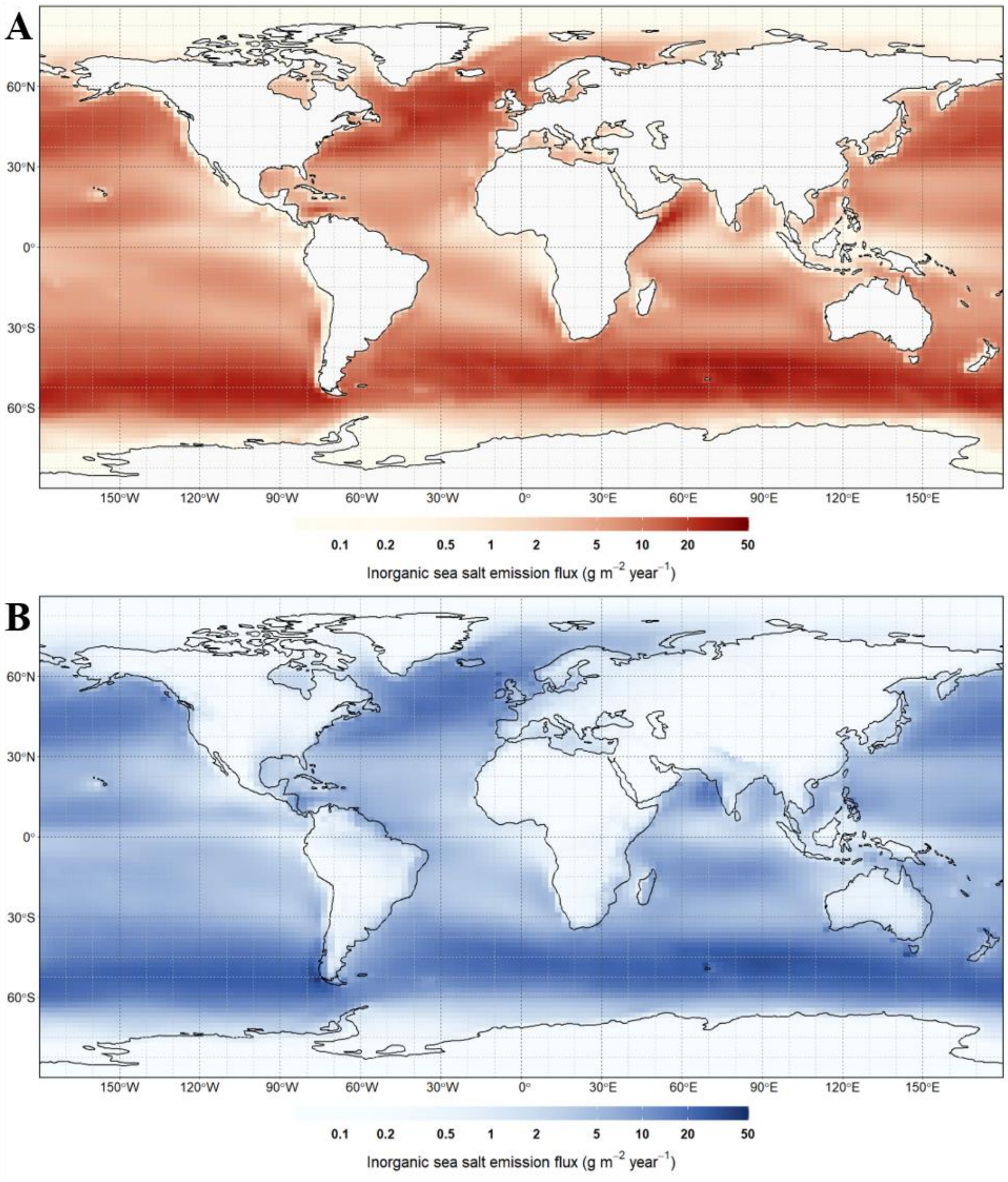

fig. S5.

Spatial distributions of inorganic sea salt emission flux (A) and total deposition flux (B). The total deposition flux includes both dry deposition and wet deposition.

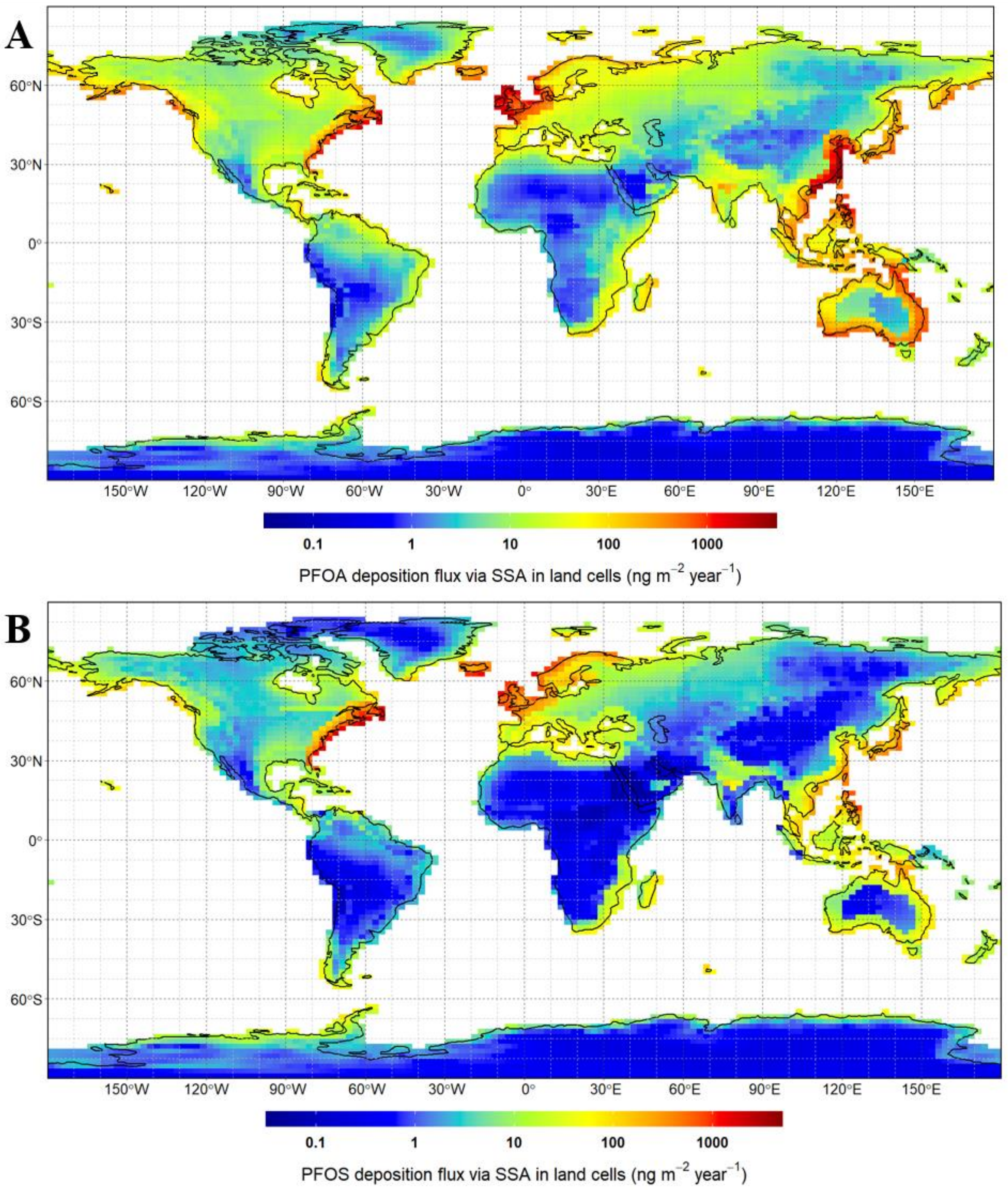

fig. S6.

PFAAs deposition flux in land cells following the emission and transport on SSA. Panel A is for PFOA and Panel B for PFOS. Wet and dry deposition fluxes are combined.

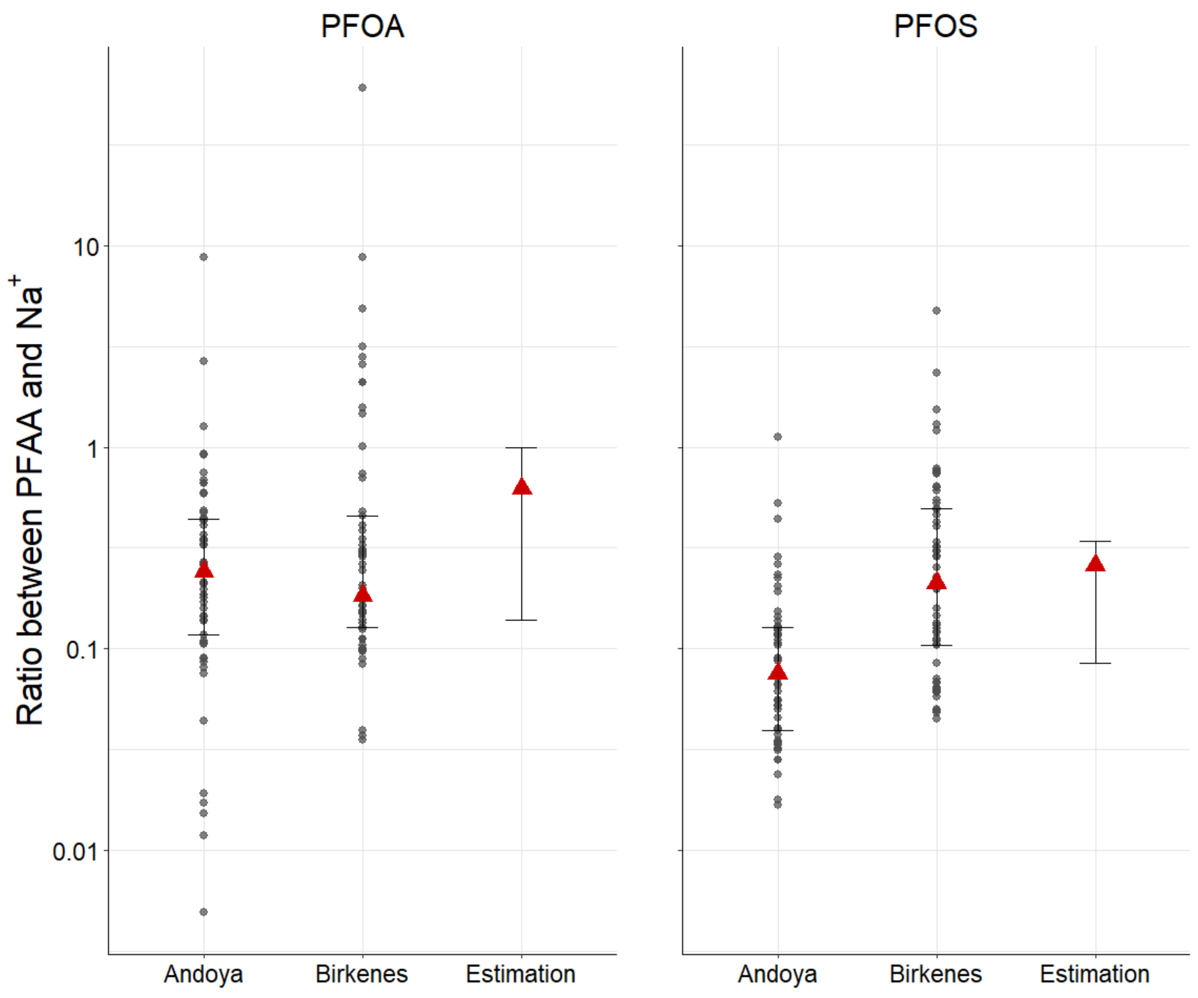

fig. S7.

Comparison between PFAA/Na^+ ratios calculated based on the SSA column burden and the PFAA/Na^+ ratios of aerosol samples from Andøya and Birkenes. The black points indicate the ratios in the aerosol samples. For the aerosol samples, the red points and the extended upper and lower hingers represent the median, the 1st quantile and the 3rd quantile of the ratios. The red points and extended upper and lower hingers for the estimation represent the results in the mean, low and high scenarios. The estimated ratios at the two locations are the same.

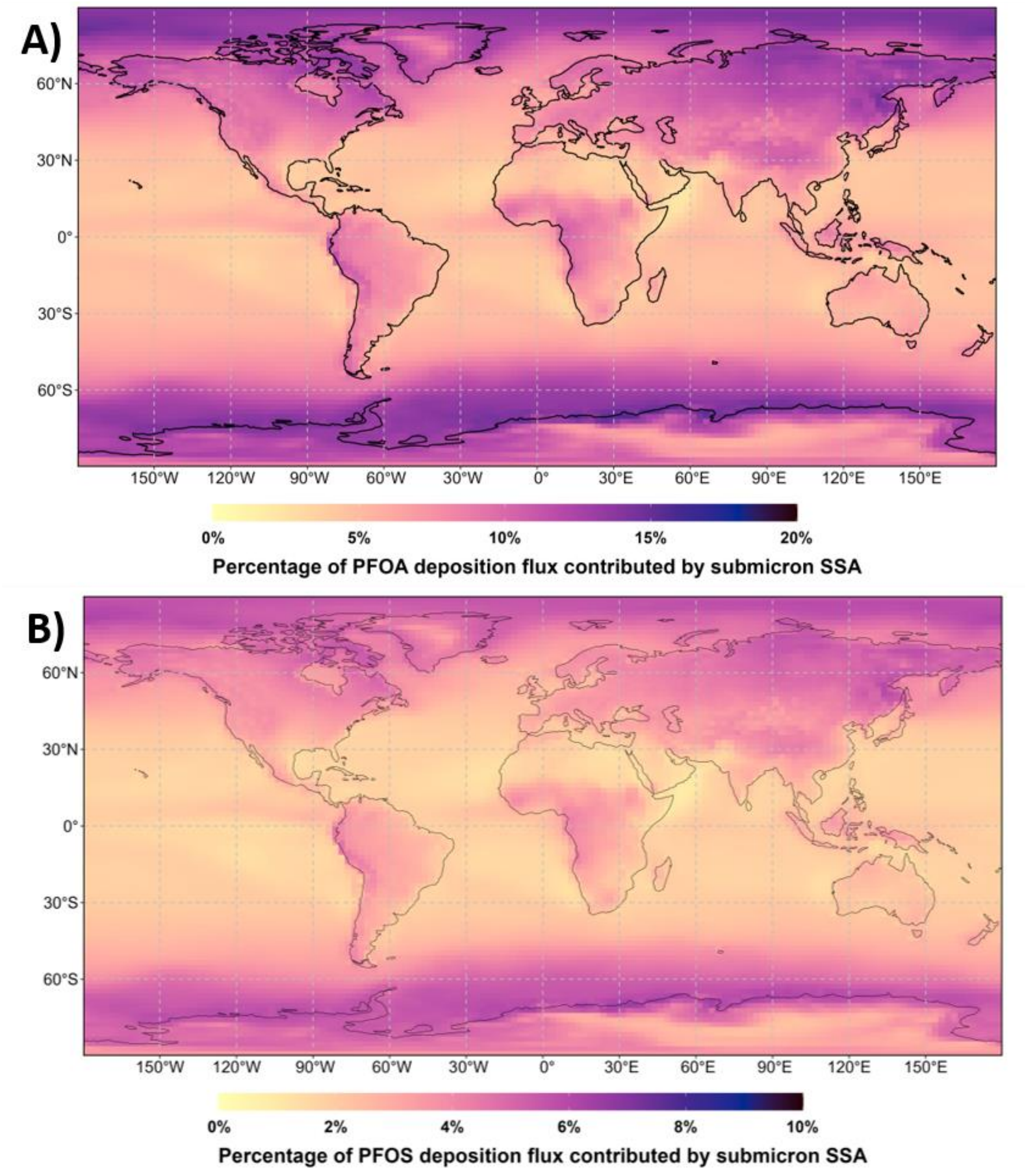

fig. S8.

Contribution of submicron SSA to and total deposition flux of PFOA (A) and PFOS (B) on SSA.

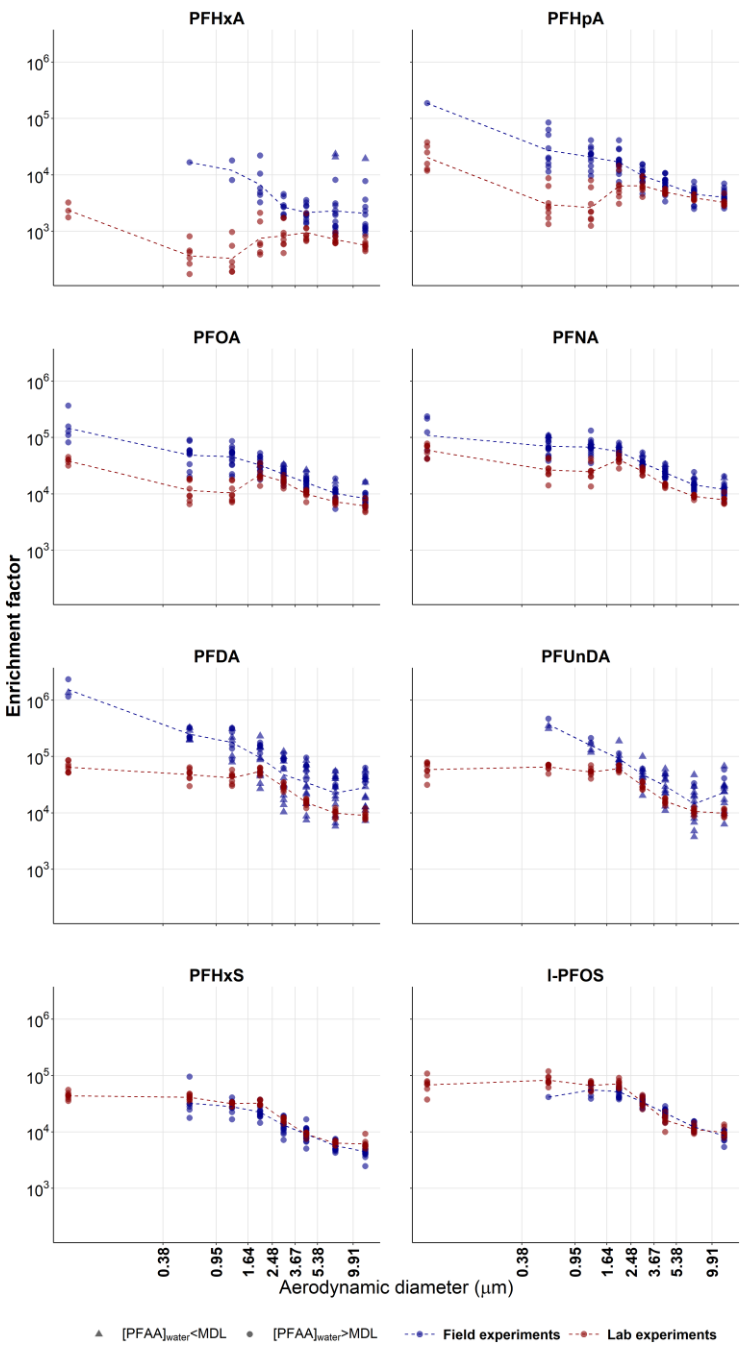

fig. S9.

Enrichment factors derived from sea spray chamber experiments conducted in the laboratory (*16*) and the field (this study) as a function of particle size. The laboratory experiments include nine experiments with expected concentrations of PFAAs between 0.3 – 20 ng L^{-1} and only data above the MDL are included. For the field experiments, EFs calculated using MDL_{water} are indicated by triangular markers. The dashed lines are provided to guide the eye and connect the geometric mean EF for each size fraction.

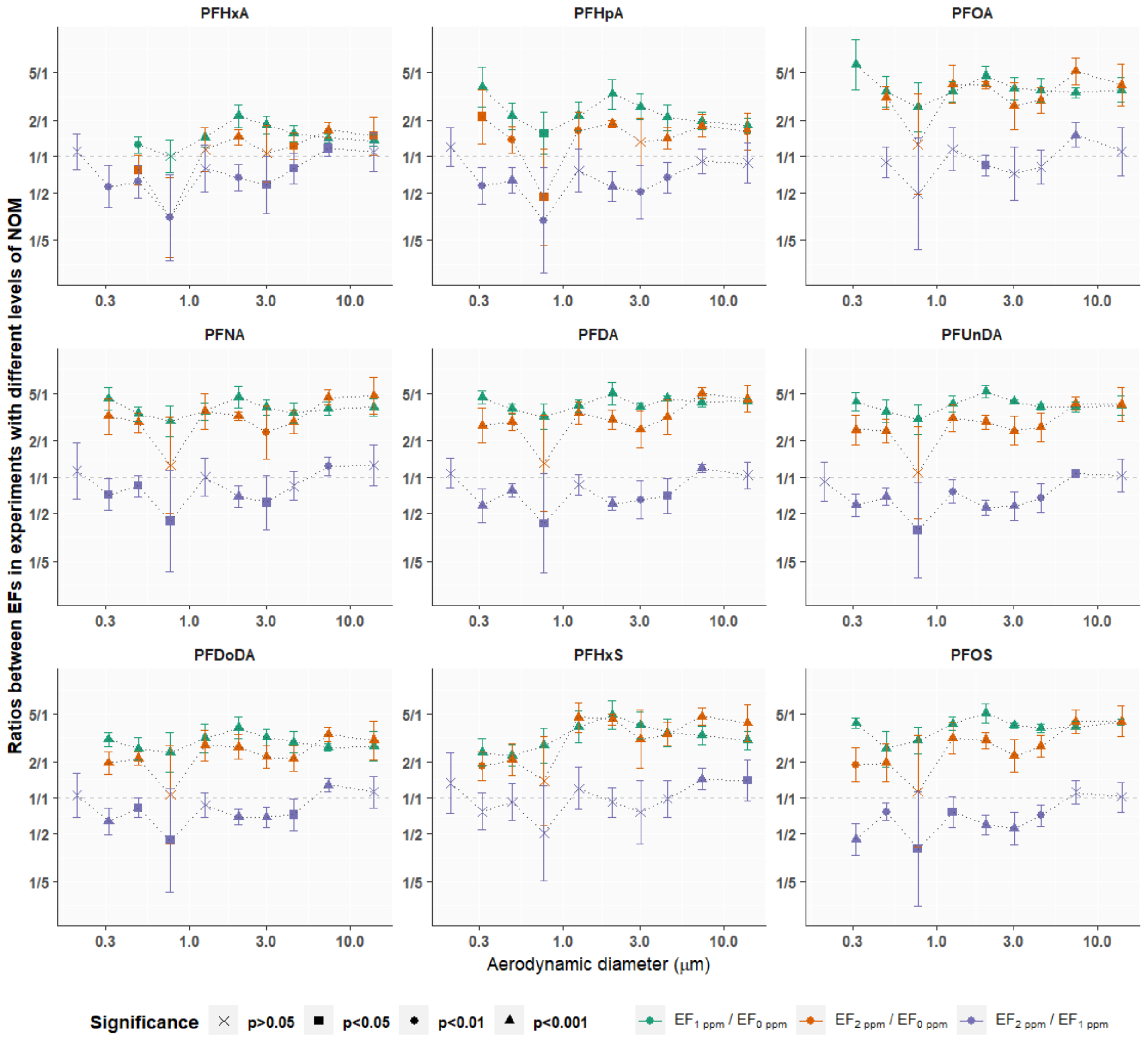

fig. S10.

Ratios between the EFs in experiment groups with different levels of TOC. $EF_{0\ ppm}$, $EF_{1\ ppm}$ and $EF_{2\ ppm}$ refer to the EFs in Exp-SS, Exp-NOM1 and Exp-NOM2, respectively. The markers represent the mean ratios and the error bars indicate the standard deviations of the ratios. The shapes of the markers indicate whether the ratio of a certain PFAA in a specific size fraction is significantly different from 1 (*t*-test) at different confidence levels.

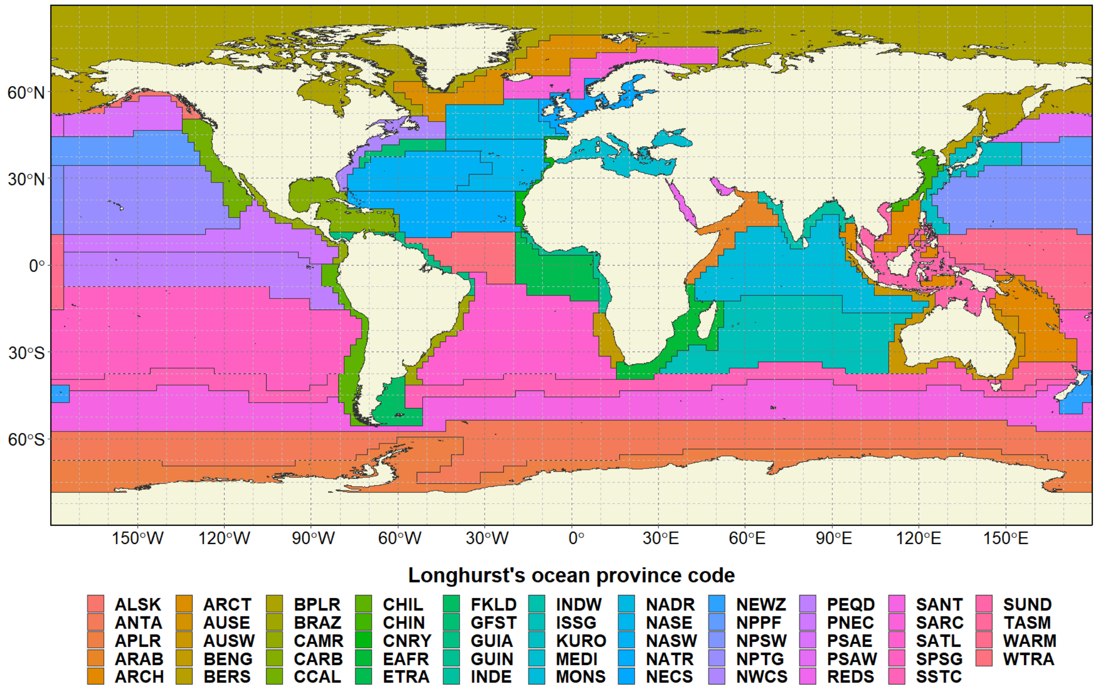

fig. S11. Longhurst's biogeochemical ocean provinces. Names and description of the ocean provinces are presented in table S4.

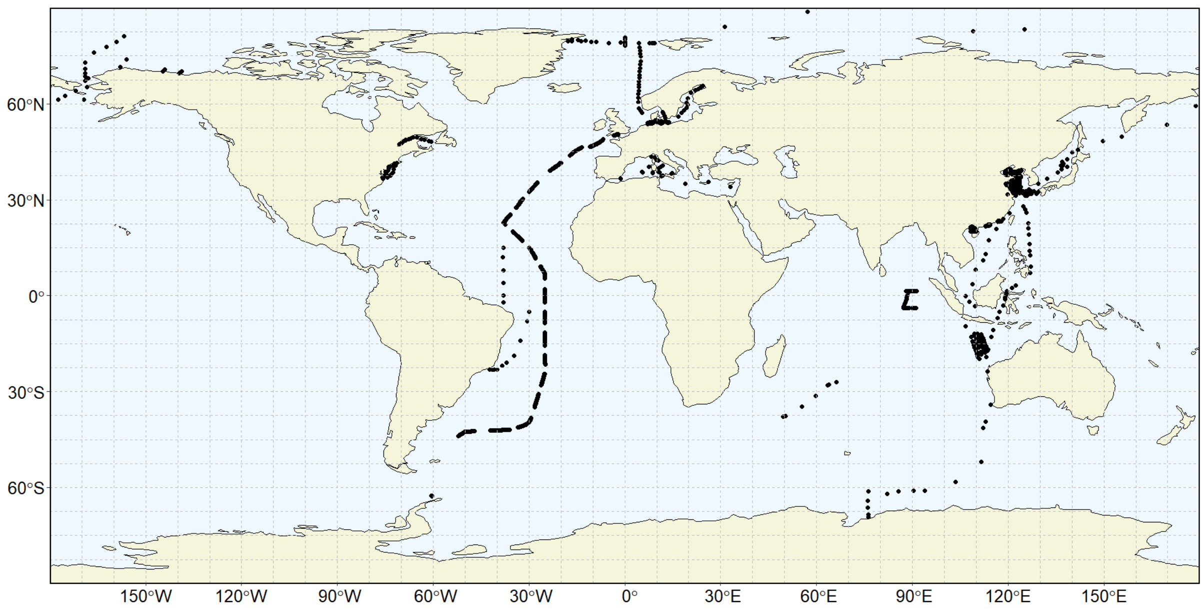

fig. S12.

Available measurements of concentrations of PFAAs in the global oceans. Only studies published after 2015 are included (table S12).

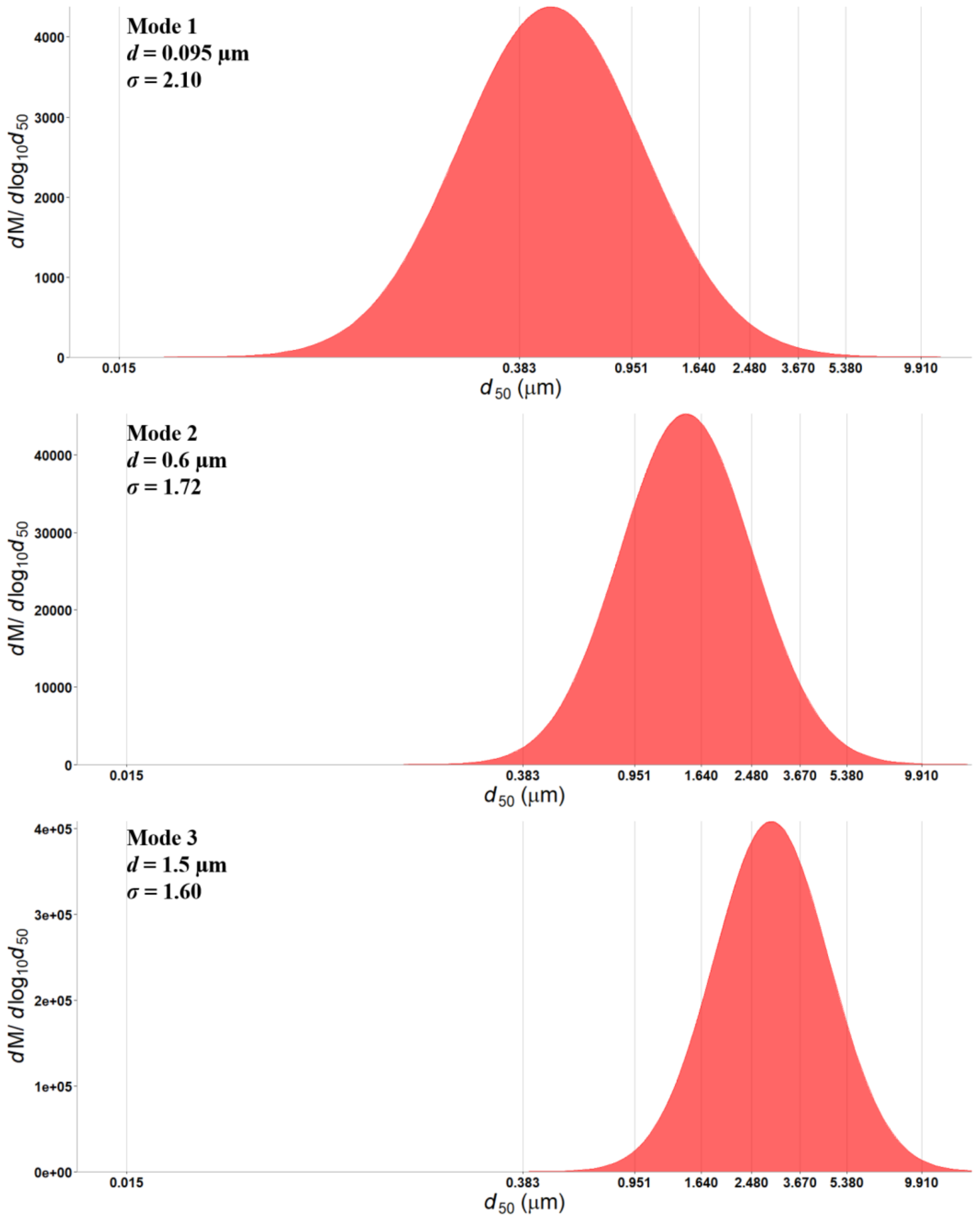

fig. S13.

Mass-size distribution of the three inorganic SSA modes in NorESM. The x-axis is the cutoff size of the cascade impactor. The shaded area indicates the mass of SSA in the eight size fractions.

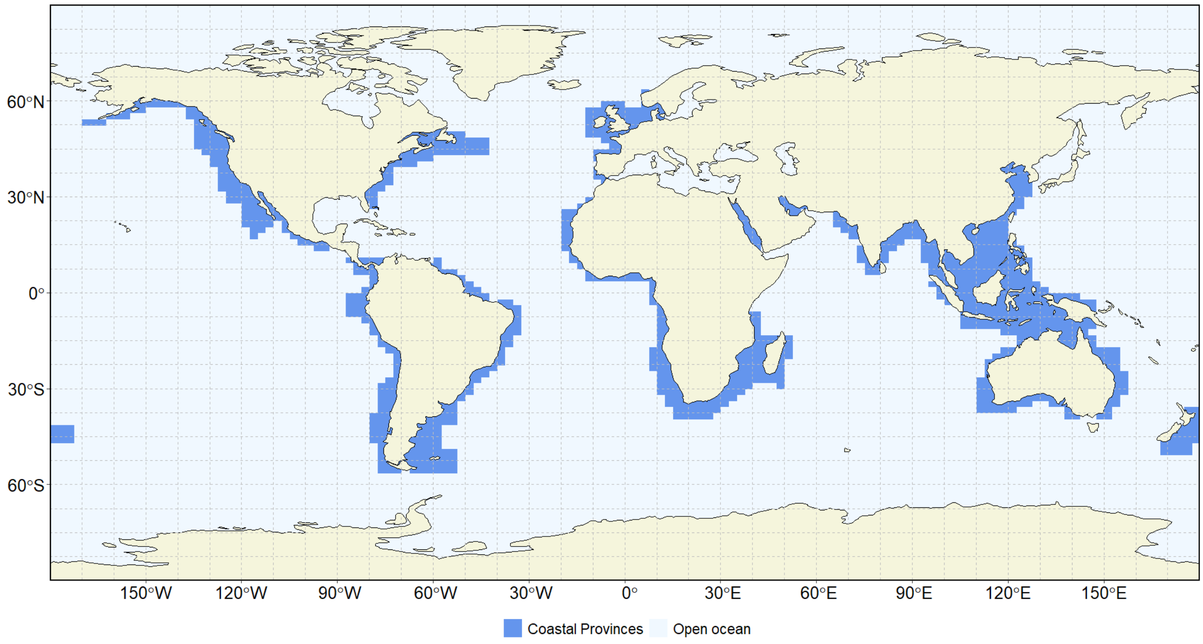

fig. S14.

Coverage of coastal Longhurst provinces.

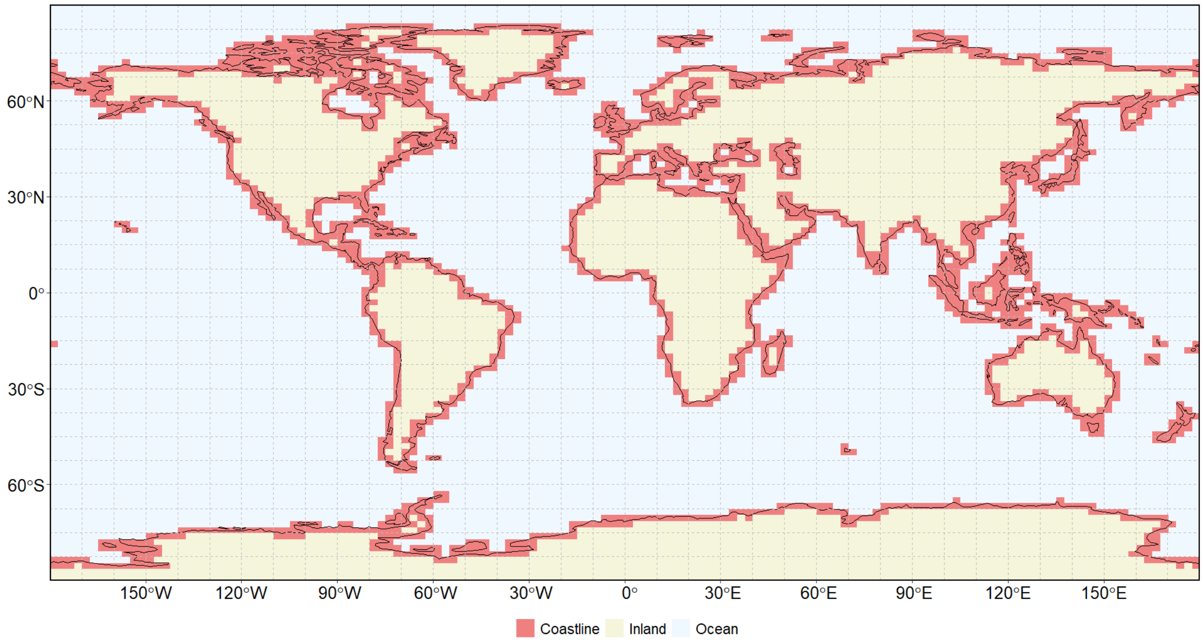

fig. S15.

Coverage of inland cells and coastline cells.

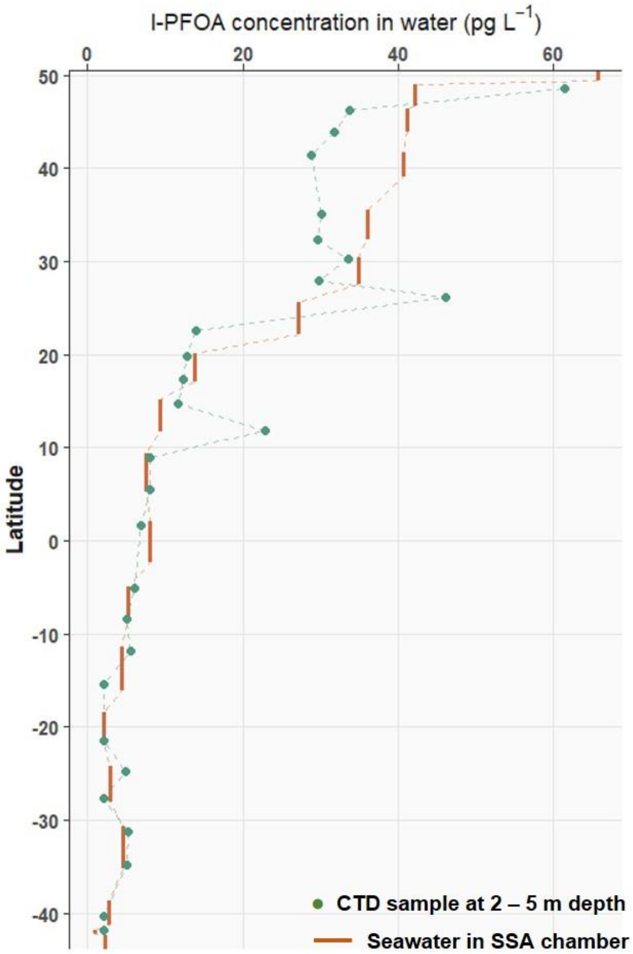

fig S16.

Comparison between l-PFOA concentrations in the chamber water and in the CTD surface samples (2 – 5 m depth, 5 L of seawater) (8) during the AMT29 cruise.

table S1.

Summary of the enrichment factors (×10^4) measured in the field experiments. MDL_{water} is used in the calculation if the compound was not detected in the seawater.

		0.02–0.38 µm	0.38–0.95 µm	0.95– 1.6 µm	1.6– 2.5 µm	2.5– 3.7 µm	3.7– 5.4 µm	5.4– 9.9 µm	>9.9 µm
PFHxA	Median		1.7	1.3	0.53	0.24	0.2	0.17	0.12
	Min-Max			0.81 – 1.8	0.32 – 2.2	0.18 – 0.45	0.14 – 0.36	0.082 – 2.3	0.089 – 1.9
	Gmean[a]		1.7	1.2	0.67	0.27	0.21	0.23	0.21
	GSD[a]			0.25	0.3	0.15	0.14	0.46	0.4
	Slope[b]				0.64	0.27	0.22***	0.13***	0.12***
	95% CI[b]				0.4 – 0.88	0.21 – 0.33	0.16 – 0.29	0.11 – 0.15	0.1 – 0.15
PFHpA	Median	19	2.0	2.3	1.6	0.92	0.73	0.47	0.39
	Min-Max		1.1 – 8.5	0.96 – 4.1	0.75 – 4.1	0.46 – 1.5	0.34 – 1.1	0.25 – 0.76	0.25 – 0.7
	Gmean	18.7	2.7	2.1	1.7	0.96	0.7	0.45	0.4
	GSD		0.31	0.18	0.18	0.13	0.13	0.12	0.11
	Slope		2.7	2.4***	1.7***	1.1***	0.8***	0.45***	0.4***
	95% CI		1.1 – 4.3	1.9 – 2.8	1.5 – 1.9	0.9 – 1.2	0.62 – 0.98	0.39 – 0.51	0.34 – 0.47
PFOA	Median	13	5.4	5.2	3.4	2.3	1.6	1.1	0.79
	Min-Max	8.2 – 37	1.7 – 9	2 – 8.6	1.8 – 5.3	1.4 – 3.3	1.1 – 2.7	0.54 – 1.9	0.53 – 1.6
	Gmean	14.6	4.9	4.5	3.2	2.2	1.6	1	0.8
	GSD	0.25	0.21	0.17	0.13	0.11	0.11	0.13	0.13
	Slope	12.9	5.1***	5.2***	3.5***	2.1***	1.5***	0.82***	0.68***
	95% CI	7.3 – 18.6	3.4 – 6.9	4.2 – 6.1	3 – 4.1	1.9 – 2.2	1.2 – 1.8	0.68 – 0.96	0.56 – 0.81
PFNA	Median	12	6.6	6.9	5.5	3.6	2.3	1.4	1.2
	Min-Max	4.2 – 24	3.8 – 11	3.5 – 13	4.2 – 8	2.8 – 5.4	1.9 – 3.5	0.86 – 2.4	0.73 – 2.1
	Gmean	10.9	7	6.8	5.7	3.6	2.4	1.4	1.2
	GSD	0.34	0.14	0.12	0.08	0.08	0.08	0.1	0.11
	Slope	9.7	5.4***	6.4***	5.3***	3.2***	2.2***	1.2***	1***
	95% CI	3.2 – 16.2	4.6 – 6.3	5.6 – 7.2	5.1 – 5.6	3 – 3.4	1.8 – 2.6	1.1 – 1.3	0.91 – 1.1
PFDA	Median	140	23	19	13	6.1	4.2	2.9	3.8
	Min-Max	120 – 230	19 – 33	8 – 32	2.7 – 23	1 – 12	0.74 – 9.5	0.58 – 5.5	0.73 – 6.3
	Gmean	154.3	24.8	17.8	9.5	4.8	3.5	2.3	2.8
	GSD	0.16	0.09	0.23	0.29	0.34	0.34	0.31	0.28
	Slope		26.2***	24*	14.2**	8.3**	6.3**	3.6**	4.3**
	95% CI		21.2 – 31.1	16.4 – 31.5	12.2 – 16.2	6.9 – 9.6	4.5 – 8.1	2.9 – 4.3	3.6 – 4.9
PFUnDA	Median		35	17	8.7	5	3.4	1.6	2.6
	Min-Max		31 – 47	12 – 21	6.1 – 19	2 – 10	1.1 – 6.1	0.38 – 4.7	0.63 – 6.8
	Gmean		37.2	15.7	9.1	4.8	3	1.5	2.3
	GSD		0.09	0.1	0.15	0.18	0.23	0.32	0.29
	Slope			15.6	10.1	5.4	4	2.4	3.8
	95% CI			12 – 20	6.9 – 13.2	3.9 – 7	3.1 – 4.9	1.6 – 3.2	2.4 – 5.1
PFHxS	Median		3	2.9	2.1	1.3	0.89	0.55	0.46
	Min-Max		1.8 – 9.6	1.7 – 4.1	1.5 – 3.7	0.72 – 1.9	0.51 – 1.7	0.43 – 0.75	0.25 – 0.59
	Gmean		3.2	2.8	2.2	1.3	0.91	0.57	0.45
	GSD		0.25	0.12	0.1	0.12	0.12	0.08	0.09
	Slope		2.6	2.8***	2.2***	1.4***	1***	0.57***	0.43***
	95% CI		2 – 3.3	2.4 – 3.2	1.7 – 2.7	1.2 – 1.7	0.6 – 1.4	0.5 – 0.63	0.27 – 0.58
l-PFOS	Median		4.1	5.4	5.6	3.3	2.2	1.2	0.88
	Min-Max			3.9 – 7.3	3.8 – 6.6	2.5 – 4.3	1.5 – 2.9	0.99 – 1.5	0.54 – 1.1
	Gmean		4.1	5.6	5.2	3.4	2.2	1.2	0.86
	GSD			0.1	0.08	0.06	0.07	0.06	0.08
	Slope			6.5***	5.9***	3.3***	2***	1.1***	0.7***
	95% CI			5.6 – 7.5	5.7 – 6	2.8 – 3.9	1.5 – 2.6	0.83 – 1.3	0.25 – 1.1
br-PFOS	Median		2.8	4.8	5.1	3.2	2	1.2	0.81
	Min-Max		1.8 – 4.1	2.1 – 7.2	1.5 – 6.9	0.99 – 3.9	0.86 – 2.5	0.48 – 1.6	0.31 – 1.1
	Gmean		2.8	4.8	4.6	3	2	1.1	0.75
	GSD		0.13	0.11	0.17	0.16	0.13	0.14	0.15
	Slope		3.5*	5.9***	5.8***	3.4***	2.1***	1.1***	0.78***
	95% CI		1.7 – 5.3	4 – 7.9	5.5 – 6.2	3 – 3.9	2 – 2.3	1.1 – 1.2	0.6 – 0.97

[a] Gmean and GSD are the geometric mean and geometric standard deviation of the EFs, respectively.

[b] Slope is estimated based on the linear regression in fig. S2 in the SM. The number of asterisks indicate the *p*-value of the correlation (* $p<0.05$, ** $p<0.01$, *** $p<0.001$). CI is 95% the confidence interval of the slope at $\alpha=0.05$.

table S2.

Coefficients of the log-log linear relationship between the slopes of >0.95 µm size fractions in fig. S2 and particle size, i.e. $\log_{10}(EF_{slope}) = b \cdot \log_{10}(d_p) + constant$.

	b	SE_b	constant	$SE_{constant}$	r^2
PFHxA	-1.05	0.16	4.09	0.11	0.92
PFHpA	-0.84	0.07	4.45	0.04	0.98
PFOA	-0.95	0.06	4.80	0.04	0.98
PFNA	-0.90	0.07	4.93	0.05	0.97
PFDA	-0.82	0.13	5.38	0.09	0.91
PFHxS	-0.88	0.05	4.57	0.03	0.99
l-PFOS	-1.07	0.09	5.00	0.06	0.98
br-PFOS	-0.99	0.10	4.97	0.06	0.96

table S3.

Enrichment factors used in the mean (low – high) emission scenario for the three SSA modes in NorESM2.

	Enrichment factor (×10^4)	
	PFOA	PFOS
Mode 1 (0.095 µm)	6.0 (3.6 – 11.3)	4.5 (3.1 – 5.9)
Mode 2 (0.6 µm)	3.6 (2.9 – 4.3)	4.8 (4.1 – 5.5)
Mode 3 (1.5 µm)	2.0 (1.7 – 2.3)	3.1 (2.8 – 3.3)

table S4.

Longhurst ocean provinces and the PFAA concentrations used in the estimation of PFAA emission from the global oceans.

Code	Province name	Ocean	Biome	No. of data[a]	Ref. province[b]	PFOA Q1[c]	PFOA Median	PFOA Q3[c]	PFOS Q1[c]	PFOS Median	PFOS Q3[c]
ANTA	Antarctic	Antarctic	Polar	6		16	16	24	13	13	14
APLR	Austral polar	Antarctic	Polar	4		15	16	16	13	14	15
SSTC	South subtropical convergence	Antarctic	Westerly	13	SANT SSTC	2.6	2.9	8.4	1.9	3.1	3.5
SANT	Subantarctic water ring	Antarctic	Westerly	1	SANT SSTC	2.6	2.9	8.4	1.9	3.1	3.5
BPRL	Boreal polar	Arctic	Polar	27		42	63	77	3.9	14	37
NECS	Northeast Atlantic shelves	Atlantic	Coastal	11		71	260	340	29.2	78	93
CNRY	Canary current coast	Atlantic	Coastal		NASE NASW NATR	15	34	40	5.3	8	9.8
GUIN	Guinea current coast	Atlantic	Coastal		WTRA	6.6	8.4	8.8	1.5	1.8	1.8
GUIA	Guianas coast	Atlantic	Coastal	5		3	7.1	11	0.6	0.6	0.6
NWCS	Northwest Atlantic shelves	Atlantic	Coastal	11		164	218	306	107	210	320
BRAZ	Brazilian current coast	Atlantic	Coastal	8		3	7.1	11	0.6	0.6	0.6
FKLD	Southwest Atlantic shelves	Atlantic	Coastal		SANT SSTC	2.6	2.9	8.4	1.9	3.1	3.5
BENG	Benguela current coast	Atlantic	Coastal		SATL	3.1	4.9	5.2	1.2	1.5	1.8
CHIL	Chile-Peru Current Coastal	Atlantic	Coastal		WTRA SATL SANT SSTC	3.1	5.2	8.4	1.2	1.8	2.1
ARCT	Atlantic Arctic	Atlantic	Polar	6		42	45	54	35	41	43
SARC	Atlantic sub-Arctic	Atlantic	Polar	7		54	60	114	64	101	127
NATR	North Atlantic tropical gyral	Atlantic	Trade wind	13		10	15	30	3.8	5.3	7.1
WTRA	Western tropical Atlantic	Atlantic	Trade wind	16		6.6	8.4	8.8	1.5	1.8	1.8

Code	Province name	Ocean	Biome	No. of data[a]	Ref. province[b]	PFOA			PFOS		
						Q1[c]	Median	Q3[c]	Q1[c]	Median	Q3[c]
ETRA	Eastern tropical Atlantic	Atlantic	Trade wind		WTRA	6.6	8.4	8.8	1.5	1.8	1.8
SATL	South Atlantic gyral	Atlantic	Trade wind	25		3.1	4.9	5.2	1.2	1.5	1.8
CARB	Caribbean	Atlantic	Trade wind		NATR	10	15	30	3.8	5.3	7.1
NADR	North Atlantic Drift	Atlantic	Westerly	5		45	45	48	10	10	11
GFST	Gulf Stream	Atlantic	Westerly		NASW	40	40	40	9.8	9.8	9.8
NASW	Northwest Atlantic subtropical gyral	Atlantic	Westerly	3		40	40	40	9.8	9.8	9.8
MEDI	Mediterranean Sea	Atlantic	Westerly	14		48	56	79	37	44	46
NASE	Northeast Atlantic subtropical gyral	Atlantic	Westerly	10		38	42	45	8.8	10	11
EAFR	East African coast	Indian	Coastal			10	23	29	9	9	9
REDS	Red Sea, Persian Gulf	Indian	Coastal		MONS	13	17	25	0.8	0.8	1.5
INDE	Eastern India coast	Indian	Coastal		MONS	13	17	25	0.8	0.8	1.5
INDW	Western India coast	Indian	Coastal		MONS	13	17	25	0.8	0.8	1.5
AUSW	Western Australian and Indonesian coast	Indian	Coastal	4		57	167	438	9.8	13	13.3
MONS	Indian monsoon gyre	Indian	Trade wind	17		13	17	25	0.8	0.8	1.5
ISSG	Indian South subtropical gyre	Indian	Trade wind	9		9	15.9	47.7	0.8	0.8	1.2
ARAB	Northwest Arabian Sea upwelling	Indian	Westerly		MONS	13	17	25	0.8	0.8	1.5
ALSK	Alaska coastal downwelling	Pacific	Coastal		BERS PSAW	81	83	83	15	16	16
CCAL	Coastal Californian current	Pacific	Coastal		NASE NASW NADR NATR	22	38	45	6.2	8.8	10
CAMR	Central American coast	Pacific	Coastal		NATR	10	15	30	3.8	5.3	7.1

Code	Province name	Ocean	Biome	No. of data[a]	Ref. province[b]	PFOA Q1[c]	PFOA Median	PFOA Q3[c]	PFOS Q1[c]	PFOS Median	PFOS Q3[c]
CHIN	China Sea	Pacific	Coastal	22		1074	2136	4075	60	80	108
SUND	Sunda-Arafura shelves	Pacific	Coastal	18		62	157	384	2.9	38	138
AUSE	East Australian coast	Pacific	Coastal		AUSW	57	167	438	9.8	13	13
NEWZ	New Zealand coast	Pacific	Coastal		SANT SSTC	2.6	2.9	8.4	1.9	3.1	3.5
BERS	North Pacific epicontinental sea	Pacific	Polar	11	BERS PSAW	81	83	83	15	16	16
PSAE	Eastern Pacific subarctic gyres	Pacific	Westerly		BERS PSAW	81	83	83	15	16	16
PSAW	Western Pacific subarctic gyres	Pacific	Westerly	1	BERS PSAW	81	83	83	15	16	16
KURO	Kuroshio current	Pacific	Westerly	16		136	166	257	41	90	137
NPPF	North Pacific polar front	Pacific	Westerly		NASE NASW NADR	40	45	45	10	10	11
NPSW	Northwest Pacific subtropical	Pacific	Westerly		NASE NASW NATR	15	34	40	5	8.0	9.8
TASM	Tasman Sea	Pacific	Westerly		SATL	3.1	4.9	5.2	1.2	1.5	1.8
NPTG	North Pacific Tropical gyre	Pacific	Trade wind		NASE NASW NATR	15	34	40	5	8.0	9.8
PNEC	North Pacific equatorial counter current	Pacific	Trade wind		WTRA	6.6	8.4	8.8	1.5	1.8	1.8
PEQD	Pacific equatorial divergence	Pacific	Trade wind		WTRA	6.6	8.4	8.8	1.5	1.8	1.8
WARM	Western Pacific warm pool	Pacific	Trade wind		WTRA	6.6	8.4	8.8	1.5	1.8	1.8
ARCH	Archipelagic deep basins	Pacific	Trade wind		SATL	3.1	4.9	5.2	1.2	1.5	1.8
SPSG	South Pacific gyre	Pacific	Trade wind		SATL	3.1	4.9	5.2	1.2	1.5	1.8

[a] The number of grid cells that have field measurement of PFAAs in seawater.
[b] Field data in the reference province(s) are used to determine the PFAA concentrations in provinces with no data.
[c] Q1 and Q3 represent the 1st and the 3rd quartile of the PFAA concentrations in each province.

table S5.

Pearson correlation coefficients (*r*) between enrichment factors (log-transformed) and seawater properties. Salinity (S), water temperature (T), conductivity (C) and Fluorescence (F). The number of asterisks indicates the significance level: *$p<0.05$, **$p<0.01$ and ***$p<0.001$.

	PFHxA	PFHpA	PFOA	PFNA	PFHxS	PFOS
0.015 - 0.383 µm			n = 5<br>r_S = -0.98**	n = 5<br>r_T = 0.97**<br>r_C = 0.98**<br>r_F = -0.93*		
0.383 - 0.951 µm		n = 9<br>r_S = -0.80**<br>r_T = 0.79*<br>r_C = 0.72*<br>r_F = -0.71*	n = 11<br>r_S = -0.69*<br>r_T = 0.77**<br>r_C = 0.73*<br>r_F = -0.71*		n = 6<br>r_S = -0.95*<br>r_T = 0.83*	
0.951 - 1.64 µm						
1.64 - 2.48 µm						n = 18<br>r_S = 0.49**
2.48 - 3.67 µm		n = 17<br>r_F = -0.52*				
3.67 - 5.38 µm	n = 11<br>r_F = 0.69*					n = 18<br>r_T = 0.49*<br>r_C = 0.52*
5.38 - 9.91 µm		n = 18<br>r_T = 0.64**<br>r_C = 0.61**<br>r_F = -0.63**				n = 18<br>r_T = 0.59**<br>r_C = 0.58*<br>r_F = -0.48*
>9.91 µm		n = 18<br>r_T = 0.52*<br>r_C = 0.49*<br>r_F = -0.54*			n = 16<br>r_S = 0.53*<br>r_F = -0.61*	n = 17<br>r_S = 0.56*<br>r_T = 0.56*<br>r_C = 0.60*<br>r_F = -0.62**

table S6.

Target compounds and abbreviations.

Compound	Acronym	CAS No.	Formula
Perfluoropentanoic acid	PFPeA	2706-90-3	C4F9COOH
Perfluorohexanoic acid	PFHxA	307-24-4	C5F11COOH
Perfluoroheptanoic acid	PFHpA	375-85-9	C6F13COOH
Perfluorooctanoic acid	PFOA	335-67-1	C7F15COOH
Perfluorononanoic acid	PFNA	375-95-1	C8F17COOH
Perfluorodecanoic acid	PFDA	335-76-2	C9F19COOH
Perfluoroundecanoic acid	PFUnDA	2058-94-8	C10F21COOH
Perfluorododecanoic acid	PFDoDA	307-55-1	C11F23COOH
Perfluorobutane sulfonic acid	PFBS	375-73-5	C4F9SO3H
Perfluorohexane sulfonic acid	PFHxS	355-46-4	C6F13SO3H
Perfluorooctane sulfonic acid	PFOS	1763-23-1	C8F17SO3H

table S7.
Cutoff sizes (d_{50}) of the 14-stage cascade impactor.

Impactor stages from bottom to top		
Stage No.	Cutoff size d_{50} (μm)	Size fraction
S01	0.015	1
S02	0.0306	
S03	0.0548	
S04	0.0944	
S05	0.154	
S06	0.256	
S07	0.383	2
S08	0.605	
S09	0.951	3
S10	1.64	4
S11	2.48	5
S12	3.67	6
S13	5.38	7
S14	9.91	8

table S8.
Information about the field experiments.

Exp.	Start date (UTC)	End date (UTC)	Duration (h)	Start location		End location		Air volume (m^3)	Water volume (L)
				Lat	Lon	Lat	Lon		
Exp01	2019-10-14 08:31	2019-10-15 14:33	30.0	50.518	-2.201	49.390	-4.612	17.3	8.3
Exp02	2019-10-16 09:50	2019-10-17 16:09	30.3	48.947	-6.814	46.652	-10.936	17.5	8.2
Exp03	2019-10-18 09:10	2019-10-19 15:16	30.1	46.359	-13.377	43.914	-17.148	17.3	8.3
Exp04	2019-10-20 10:05	2019-10-21 16:10	30.1	41.711	-19.838	39.065	-23.412	17.3	12.0
Exp05	2019-10-23 09:04	2019-10-24 15:07	30.1	35.489	-27.271	32.402	-30.024	17.3	11.0
Exp06	2019-10-25 12:13	2019-10-26 19:16	30.1	30.362	-31.784	27.471	-34.069	17.3	12.2
Exp07	2019-10-27 11:04	2019-10-28 18:10	30.1	25.555	-35.675	22.149	-37.280	17.3	12.6
Exp08	2019-10-29 10:35	2019-10-30 16:31	29.9	20.100	-35.099	17.077	-31.972	17.2	12.4
Exp09	2019-10-31 10:10	2019-11-01 16:09	30.0	15.138	-29.976	11.724	-27.893	17.3	12.4
Exp10	2019-11-02 10:08	2019-11-03 16:02	29.9	9.355	-26.480	5.271	-25.005	17.2	12.6
Exp11	2019-11-04 10:27	2019-11-05 16:33	30.1	2.093	-24.999	-2.341	-24.997	17.3	12.6
Exp12	2019-11-06 10:19	2019-11-07 16:26	30.1	-4.944	-25.000	-8.632	-25.007	17.3	12.5
Exp13	2019-11-08 10:10	2019-11-09 16:10	30.0	-11.367	-25.001	-16.058	-25.009	17.3	12.0
Exp14	2019-11-10 10:11	2019-11-11 16:05	29.9	-18.504	-25.108	-21.866	-24.844	17.2	11.4
Exp15	2019-11-12 09:13	2019-11-13 15:07	29.9	-24.260	-25.070	-28.115	-26.217	17.2	11.6
Exp16	2019-11-14 09:17	2019-11-15 15:13	29.9	-30.693	-26.983	-35.201	-28.408	17.2	11.2
Exp17	2019-11-16 12:32	2019-11-17 18:29	30.0	-38.692	-29.555	-41.231	-33.447	17.3	10.1
Exp18	2019-11-18 08:43	2019-11-19 14:36	29.9	-41.901	-35.431	-42.196	-42.351	17.2	10.2
Exp19	2019-11-20 09:52	2019-11-21 16:05	30.2	-42.380	-46.993	-43.958	-52.290	17.4	10.4

table S9.
MDLs, MQLs and field blanks of PFAAs in SSA and chamber water.

	SSA (pg per SPE cartridge)			Chamber water (pg per SPE cartridge)		
	MDL	MQL	Field blank (Mean±sd)	MDL	MQL	Field blank (Mean±sd)
PFHxA	3.1	10	4.7±1.0	3.4	11	19±1
PFHpA	1.2	4.0	0.9±0.4	3.3	11	11±1
PFOA	1.5	5.2	2.9±0.5	9.7	32	91±3
PFNA	0.3	1.1	0.6±0.1	5.2	17	12±2
PFDA	0.5	1.7	0.8±0.2	5.1	17	12±2
PFUnDA	1.3	4.3	1.4±0.4	6.4	21	9.2±2.1
PFDoDA	2.5	8.3	0.4±0.8	6.8	23	4.5±2.3
PFBS	3.4	11	5.5±1.1	1.3	4.2	1.7±0.4
PFHxS	0.9	3.2	1.4±0.3	4.6	15	3.3±1.5
l-PFOS	1.8	6.0	0.9±0.6	2.7	9	2.2±0.9
br-PFOS	0.3	1.1	0.3±0.1	3.9	13	1.4±1.3

table S10.

Concentrations (median, min and max) of PFAAs, detection frequencies (%) in chamber water and the percentage above the MQLs.

	Median (pg L^{-1})	Min – Max (pg L^{-1})	Detection frequency	>MQL
PFHxA	6.1	<MDL – 35	79%	79%
PFHpA	5.0	<MDL – 32	100%	95%
PFOA	8.0	<MDL – 66	95%	75%
PFNA	3.1	<MDL – 18	91%	74%
PFDA	<MDL	<MDL – 1.3	33%	0%
PFUnDA	<MDL	<MDL – 0.9	12%	0%
PFDoDA	<MDL	<MDL	0%	0%
PFBS	0.8	<MDL – 13	53%	53%
PFHxS	1.6	0.6 – 22	100%	63%
l-PFOS	1.2	0.5 – 9.2	100%	100%
br-PFOS	2.0	0.8 – 21	100%	47%

table S11.

Size-resolved detection frequency of PFAAs in SSA and percentage above the MQLs.

	0.02-0.38 (μm)	0.38-0.95 (μm)	0.95-1.6 (μm)	1.6-2.5 (μm)	2.5-3.7 (μm)	3.7-5.4 (μm)	5.4-9.9 (μm)	>9.91 (μm)
Detection frequency								
PFHxA	5%	11%	16%	37%	47%	63%	89%	74%
PFHpA	5%	53%	68%	89%	95%	100%	100%	100%
PFOA	37%	63%	89%	100%	100%	100%	100%	100%
PFNA	37%	95%	100%	100%	100%	100%	100%	100%
PFDA	26%	58%	63%	89%	100%	100%	100%	95%
PFUnDA	5%	21%	32%	53%	58%	68%	84%	79%
PFDoDA	5%	5%	5%	5%	16%	21%	58%	68%
PFHxS	0%	32%	42%	74%	95%	100%	95%	84%
l-PFOS	0%	5%	37%	100%	100%	100%	100%	89%
br-PFOS	0%	26%	84%	100%	100%	100%	100%	100%
Percentage >MQL								
PFHxA	0%	0%	0%	0%	5%	26%	37%	42%
PFHpA	0%	11%	42%	63%	79%	95%	95%	84%
PFOA	0%	53%	53%	68%	95%	95%	95%	89%
PFNA	5%	74%	89%	100%	100%	100%	100%	100%
PFDA	5%	21%	42%	58%	68%	84%	95%	84%
PFUnDA	5%	5%	5%	11%	32%	47%	53%	68%
PFDoDA	0%	5%	5%	0%	0%	0%	11%	32%
PFHxS	0%	5%	26%	32%	42%	58%	79%	68%
l-PFOS	0%	0%	21%	32%	63%	79%	89%	68%
br-PFOS	0%	5%	37%	84%	100%	100%	100%	79%

table S12.

Studies used in the determination of PFOA and PFOS concentrations for each Longhurst ocean province. Only studies after 2015 are included.

Study	Reference No.	Region
This study	-	Atlantic Ocean
Brumovský *et al*. 2016	(*50*)	Mediterranean Sea
Casas *et al*. 2020	(*28*)	Antarctica coast
Diao *et al*. 2022	(*51*)	South China Sea
Feng *et al*. 2020	(*52*)	Yellow Sea
Han *et al*. 2022	(*53*)	Indian Ocean
Hope 2020	(*54*)	East coast of Africa
Joerss *et al*. 2019	(*55*)	Atlantic Ocean and the Baltic Sea
Joerss *et al*. 2020	(*56*)	Arctic Ocean
Li *et al.* 2018	(*57*)	Arctic Ocean and North Pacific Ocean
Miranda *et al*. 2021	(*58*)	Atlantic Ocean, east coast of Brazil
Nguyen *et al*. 2017	(*59*)	The Baltic Sea
Picard *et al*. 2021	(*60*)	East coast of North America
Shan *et al*. 2021	(*61*)	Pacific and the Southern Ocean
Wang *et al*. 2019	(*62*)	South China Sea
Xiao *et al*. 2021	(*63*)	South China Sea
Yamazaki *et al.* 2019	(*64*)	Japan Sea and the Mediterranean Sea
Yeung *et al*. 2017	(*65*)	Arctic Ocean
Zhang *et al*. 2019	(*66*)	Atlantic Ocean
Zhao *et al*. 2017	(*67*)	Bohai Sea and Yellow Sea

Table S13.

Conditions of the complementary laboratory experiments.

Experiment group	Expected PFAA concentration	Salt type	Salinity	TOC level	Water temperature	RH[a] at inlet	Duration
Exp-NaCl	~3 ng L^{-1}	NaCl	37 psu	0	15°C	-[b]	24 h
Exp-SS	~3 ng L^{-1}	Artificial sea salt	37 psu	0	15°C	~58%	24 h
Exp-NOM1	~3 ng L^{-1}	Artificial sea salt	37 psu	~1 ppm	15°C	~58%	24 h
Exp-NOM1	~3 ng L^{-1}	Artificial sea salt	37 psu	~2 ppm	15°C	~58%	24 h

[a]RH is the relative humidity.

[b]Not measured due to technique problem with the RH sensor.

Data S1. (separate file)

PFAA and Na^+ concentrations in SSA and in water in the field experiments.

Data S2. (separate file)

PFAA and Na^+ concentrations in SSA and in water in the complementary laboratory experiments.

REFERENCES AND NOTES

1. J. Glüge, M. Scheringer, I. T. Cousins, J. C. DeWitt, G. Goldenman, D. Herzke, R. Lohmann, C. A. Ng, X. Trier, Z. Wang, An overview of the uses of per- and polyfluoroalkyl substances (PFAS). *Environ. Sci. Process. Impacts* **22**, 2345–2373 (2020).

2. I. T. Cousins, J. H. Johansson, M. E. Salter, B. Sha, M. Scheringer, Outside the safe operating space of a new planetary boundary for per- and polyfluoroalkyl substances (PFAS). *Environ. Sci. Technol.* **56**, 11172–11179 (2022).

3. Z. Wang, I. T. Cousins, M. Scheringer, R. C. Buck, K. Hungerbühler, Global emission inventories for C4–C14 perfluoroalkyl carboxylic acid (PFCA) homologues from 1951 to 2030, Part I: Production and emissions from quantifiable sources. *Environ. Int.* **70**, 62–75 (2014).

4. Z. Wang, J. M. Boucher, M. Scheringer, I. T. Cousins, K. Hungerbühler, Toward a comprehensive global emission inventory of C4–C10 perfluoroalkanesulfonic acids (PFSAs) and related precursors: Focus on the life cycle of C8-based products and ongoing industrial transition. *Environ. Sci. Technol.* **51**, 4482–4493 (2017).

5. M. Land, C. A. de Wit, A. Bignert, I. T. Cousins, D. Herzke, J. H. Johansson, J. W. Martin, What is the effect of phasing out long-chain per- and polyfluoroalkyl substances on the concentrations of perfluoroalkyl acids and their precursors in the environment? A systematic review. *Environ. Evid.* **7**, 4 (2018).

6. I. T. Cousins, J. C. DeWitt, J. Glüge, G. Goldenman, D. Herzke, R. Lohmann, C. A. Ng, M. Scheringer, Z. Wang, The high persistence of PFAS is sufficient for their management as a chemical class. *Environ. Sci. Process. Impacts* **22**, 2307–2312 (2020).

7. K. Prevedouros, I. T. Cousins, R. C. Buck, S. H. Korzeniowski, Sources, fate and transport of perfluorocarboxylates. *Environ. Sci. Technol.* **40**, 32–44 (2006).

8. E. K. Savvidou, B. Sha, M. E. Salter, I. T. Cousins, J. H. Johansson, Horizontal and vertical distribution of perfluoroalkyl acids (PFAAs) in the water column of the Atlantic Ocean. *Environ. Sci. Technol. Lett.* **10**, 418–424 (2023).

9. N. Trilla, J. Dachs, J. Iriarte, N. Berrojalbiz, G. Casas, P. Colomer-Vidal, O. Garcia-Garin, M. vila-Costa, B. Jimenez, Perfluoroalkyl substances in the atlantic and southern oceans: forever around us, in *SETAC Europe 33rd Annual Meeting* (SETAC, 2023).

10. B. Sha, J. H. Johansson, P. Tunved, P. Bohlin-Nizzetto, I. T. Cousins, M. E. Salter, Sea spray aerosol (SSA) as a source of perfluoroalkyl acids (PFAAs) to the atmosphere: Field evidence from long-term air monitoring. *Environ. Sci. Technol.* **56**, 228–238 (2022).

11. E. D. Amato, F. Béen, *Occurrence of PFAS in sea-spray aerosol from the Dutch coast,* in *SETAC Europe 33rd Annual Meeting* (SETAC, 2023).

12. NIRAS, *Screening af Forekomsten af PFAS på Naturstyrelsens Kystnære og Vestvendte Arealer i Jylland og på Sjælland* (2023).

13. S. Rygaard Lenschow, *Can PFAS Contamination Spread to Soil and Groundwater by Aerosols and Foams Generated in the Sea Water?* (The 16th PFAS network meeting, 2022).

14. J. H. Johansson, M. E. Salter, J. C. A. Navarro, C. Leck, D. E. Nilsson, I. T. Cousins, Global transport of perfluoroalkyl acids via sea spray aerosol. *Environ. Sci. Process. Impacts* **21**, 635–649 (2019).

15. M. E. Salter, E. D. Nilsson, A. Butcher, M. Bilde, On the seawater temperature dependence of the sea spray aerosol generated by a continuous plunging jet. *J. Geophys. Res. Atmos.* **119**, 9052–9072 (2014).

16. B. Sha, J. H. Johansson, J. P. Benskin, I. T. Cousins, M. E. Salter, Influence of water concentrations of perfluoroalkyl acids (PFAAs) on their size-resolved enrichment in nascent sea spray aerosols. *Environ. Sci. Technol.* 9489–9497 (2021).

17. A. Kirkevåg, A. Grini, D. Olivié, Ø. Seland, K. Alterskjær, M. Hummel, I. H. H. Karset, A. Lewinschal, X. Liu, R. Makkonen, I. Bethke, J. Griesfeller, M. Schulz, T. Iversen, A production-tagged aerosol module for Earth system models, OsloAero5.3—Extensions and updates for CAM5.3-Oslo. *Geosci. Model Dev.* **11**, 3945–3982 (2018).

18. Ø. Seland, M. Bentsen, D. Olivié, T. Toniazzo, A. Gjermundsen, L. S. Graff, J. B. Debernard, A. K. Gupta, Y.-C. He, A. Kirkevåg, J. Schwinger, J. Tjiputra, K. S. Aas, I. Bethke, Y. Fan, J. Griesfeller, A. Grini, C. Guo, M. Ilicak, I. H. H. Karset, O. Landgren, J. Liakka, K. O. Moseid, A. Nummelin, C. Spensberger, H. Tang, Z. Zhang, C. Heinze, T. Iversen, M. Schulz, Overview of the Norwegian Earth System Model (NorESM2) and key climate response of CMIP6 DECK, historical, and scenario simulations. *Geosci. Model Dev.* **13**, 6165–6200 (2020).

19. S. M. Blichner, M. K. Sporre, R. Makkonen, T. K. Berntsen, Implementing a sectional scheme for early aerosol growth from new particle formation in the Norwegian Earth System Model v2: Comparison to observations and climate impacts. *Geosci. Model Dev.* **14**, 3335–3359 (2021).

20. S. Xie, Y. Lu, T. Wang, S. Liu, K. Jones, A. Sweetman, Estimation of PFOS emission from domestic sources in the eastern coastal region of China. *Environ. Int.* **59**, 336–343 (2013).

21. C. P. Thackray, N. E. Selin, C. J. Young, A global atmospheric chemistry model for the fate and transport of PFCAs and their precursors. *Environ. Sci. Processes Impacts* **22**, 285–293 (2020).

22. M. S. Shimizu, R. Mott, A. Potter, J. Zhou, K. Baumann, J. D. Surratt, B. Turpin, G. B. Avery, J. Harfmann, R. J. Kieber, R. N. Mead, S. A. Skrabal, J. D. Willey, Atmospheric deposition and annual flux of legacy perfluoroalkyl substances and replacement perfluoroalkyl ether carboxylic acids in wilmington, NC, USA, *Environ. Sci. Technol. Lett.* **8**, 366–372 (2021).

23. X. Fang, Q. Wang, Z. Zhao, J. Tang, C. Tian, Y. Yao, J. Yu, H. Sun, Distribution and dry deposition of alternative and legacy perfluoroalkyl and polyfluoroalkyl substances in the air above the Bohai and Yellow Seas, China, *Atmos. Environ.* **192**, 128–135 (2018).

24. W. F. Hartz, M. K. Björnsdotter, L. W. Y. Yeung, A. Hodson, E. R. Thomas, J. D. Humby, C. Day, I. E. Jogsten, A. Kärrman, R. Kallenborn, Levels and distribution profiles of per- and polyfluoroalkyl substances (PFAS) in a high Arctic Svalbard ice core. *Sci. Total Environ.* **871**, 161830 (2023).

25. H. M. Pickard, A. S. Criscitiello, C. Spencer, M. J. Sharp, D. C. G. Muir, A. O. De Silva, C. J. Young, Continuous non-marine inputs of per- and polyfluoroalkyl substances to the High Arctic: A multi-decadal temporal record. *Atmos. Chem. Phys.* **18**, 5045–5058 (2018).

26. Y. Shi, R. Vestergren, L. Xu, X. Song, X. Niu, C. Zhang, Y. Cai, Characterizing direct emissions of perfluoroalkyl substances from ongoing fluoropolymer production sources: A spatial trend study of Xiaoqing River, China, *Environ. Pollut.* **206**, 104–112 (2015).

27. P. Wang, Y. Lu, T. Wang, Y. Fu, Z. Zhu, S. Liu, S. Xie, Y. Xiao, J. P. Giesy, Occurrence and transport of 17 perfluoroalkyl acids in 12 coastal rivers in south Bohai coastal region of China with concentrated fluoropolymer facilities. *Environ. Pollut.* **190**, 115–122 (2014).

28. G. Casas, A. Martínez-Varela, J. L. Roscales, M. Vila-Costa, J. Dachs, B. Jiménez, Enrichment of perfluoroalkyl substances in the sea-surface microlayer and sea-spray aerosols in the Southern Ocean. *Environ. Pollut.* **267**, 115512 (2020).

29. Y. Li, X. Yu, X. Chen, J. Yin, W. Zhong, L. Zhu, Underlying mechanisms for the impacts of molecular structures and water chemistry on the enrichment of poly/perfluoroalkyl substances in aqueous aerosol. *Sci. Total Environ.* **803**, 150003 (2022).
30. D. A. Hansell, C. A. Carlson, D. J. Repeta, R. Schlitzer, Dissolved organic matter in the ocean: A controversy stimulates new insights. *Oceanography* **22**, 202–211 (2009).
31. M. L. Brusseau, S. Van Glubt, The influence of surfactant and solution composition on PFAS adsorption at fluid-fluid interfaces. *Water Res.* **161**, 17–26 (2019).
32. S. Christiansen, M. E. Salter, E. Gorokhova, Q. T. Nguyen, M. Bilde, Sea spray aerosol formation: Laboratory results on the role of air entrainment, water temperature, and phytoplankton biomass. *Environ. Sci. Technol.* **53**, 13107–13116 (2019).
33. N. Sanwlani, C. D. Evans, M. Müller, N. Cherukuru, P. Martin, Rising dissolved organic carbon concentrations in coastal waters of northwestern Borneo related to tropical peatland conversion *Sci. Adv.* **8**, eabi5688 (2022).
34. A. R. Longhurst, *Ecological Geography of the Sea* (Academic Press, ed. 2, 2007).
35. D. Muir, L. T. Miaz, Spatial and temporal trends of perfluoroalkyl substances in global ocean and coastal waters. *Environ. Sci. Technol.* **55**, 9527–9537 (2021).
36. E. R. Lewis, S. E. Schwartz, *Sea Salt Aerosol Production: Mechanisms, Methods, Measurements and Models* (AGU, 2004), vol. 152 of *Geophysical Monograph Series*.
37. J. Löfstedt Gilljam, J. Leonel, I. T. Cousins, J. P. Benskin, Is ongoing sulfluramid use in South America a significant source of perfluorooctanesulfonate (PFOS)? production inventories, environmental fate, and local occurrence. *Environ. Sci. Technol.* **50**, 653–659 (2016).

38. J. P. Benskin, M. G. Ikonomou, M. B. Woudneh, J. R. Cosgrove, Rapid characterization of perfluoralkyl carboxylate, sulfonate, and sulfonamide isomers by high-performance liquid chromatography–tandem mass spectrometry. *J. Chromatogr. A* **1247**, 165–170 (2012).
39. S. M. Blichner, M. K. Sporre, R. Makkonen, T. K. Berntsen, Implementing a sectional scheme for early aerosol growth from new particle formation in the Norwegian Earth System Model v2: comparison to observations and climate impacts. *Geosci. Model Dev.* **14**, 3335–3359 (2021).
40. G. J. Kooperman, M. S. Pritchard, S. J. Ghan, M. Wang, R. C. J. Somerville, L. M. Russell, Constraining the influence of natural variability to improve estimates of global aerosol indirect effects in a nudged version of the Community Atmosphere Model 5. *J. Geophys. Res. Atmos.* **117**, D23204 (2012).
41. Norwegian Earth System Model (NorESM), version release-noresm2.0.1, Norwegian Earth System Modeling hub; https://github.com/NorESMhub/NorESM.
42. D. M. Lawrence, R. A. Fisher, C. D. Koven, K. W. Oleson, S. C. Swenson, G. Bonan, N. Collier, B. Ghimire, L. van Kampenhout, D. Kennedy, E. Kluzek, P. J. Lawrence, F. Li, H. Li, D. Lombardozzi, W. J. Riley, W. J. Sacks, M. Shi, M. Vertenstein, W. R. Wieder, C. Xu, A. A. Ali, A. M. Badger, G. Bisht, M. van den Broeke, M. A. Brunke, S. P. Burns, J. Buzan, M. Clark, A. Craig, K. Dahlin, B. Drewniak, J. B. Fisher, M. Flanner, A. M. Fox, P. Gentine, F. Hoffman, G. Keppel-Aleks, R. Knox, S. Kumar, J. Lenaerts, L. R. Leung, W. H. Lipscomb, Y. Lu, A. Pandey, J. D. Pelletier, J. Perket, J. T. Randerson, D. M. Ricciuto, B. M. Sanderson, A. Slater, Z. M. Subin, J. Tang, R. Q. Thomas, M. Val Martin, X. Zeng, The Community Land Model Version 5: Description of new features, benchmarking, and impact of forcing uncertainty. *J. Adv. Model. Earth Syst.* **11**, 4245–4287 (2019).

43. J. W. Hurrell, J. J. Hack, D. Shea, J. M. Caron, J. Rosinski, A new sea surface temperature and sea ice boundary dataset for the community atmosphere model. *J. Climate* **21**, 5145–5153 (2008).

44. J. Gliß, A. Mortier, M. Schulz, E. Andrews, Y. Balkanski, S. E. Bauer, A. M. K. Benedictow, H. Bian, R. Checa-Garcia, M. Chin, P. Ginoux, J. J. Griesfeller, A. Heckel, Z. Kipling, A. Kirkevåg, H. Kokkola, P. Laj, P. Le Sager, M. T. Lund, C. Lund Myhre, H. Matsui, G. Myhre, D. Neubauer, T. van Noije, P. North, D. J. L. Olivié, S. Rémy, L. Sogacheva, T. Takemura, K. Tsigaridis, S. G. Tsyro, AeroCom phase III multi-model evaluation of the aerosol life cycle and optical properties using ground- and space-based remote sensing as well as surface in situ observations. *Atmos. Chem. Phys.* **21**, 87–128 (2021).

45. M. E. Salter, P. Zieger, J. C. Acosta Navarro, H. Grythe, A. Kirkevåg, B. Rosati, I. Riipinen, E. D. Nilsson, An empirically derived inorganic sea spray source function incorporating sea surface temperature. *Atmos. Chem. Phys.* **15**, 11047–11066 (2015).

46. M. Vichi, J. I. Allen, S. Masina, N. J. Hardman-Mountford, The emergence of ocean biogeochemical provinces: A quantitative assessment and a diagnostic for model evaluation. *Global Biogeochem. Cycles* **25** (2011).

47. G. de Leeuw, E. L. Andreas, M. D. Anguelova, C. W. Fairall, E. R. Lewis, C. O’Dowd, M. Schulz, S. E. Schwartz, Production flux of sea spray aerosol. *Rev. Geophys.* **49**, RG2001 (2011).

48. S. M. Burrows, O. Ogunro, A. A. Frossard, L. M. Russell, P. J. Rasch, S. M. Elliott, A physically based framework for modeling the organic fractionation of sea spray aerosol from bubble film Langmuir equilibria. *Atmos. Chem. Phys.* **14**, 13601–13629 (2014).

49. D. R. Kester, I. W. Duedall, D. N. Connors, R. M. Pytkowicz, Preparation of artificial seawater1. *Limnol. Oceanogr.* **12**, 176–179 (1967).

50. M. Brumovský, P. Karásková, M. Borghini, L. Nizzetto, Per- and polyfluoroalkyl substances in the Western Mediterranean Sea waters. *Chemosphere* **159**, 308–316 (2016).

51. J. Diao, Z. Chen, T. Wang, C. Su, Q. Sun, Y. Guo, Z. Zheng, L. Wang, P. Li, W. Liu, S. Hong, J. S. Khim, Perfluoroalkyl substances in marine food webs from South China Sea: Trophic transfer and human exposure implication. *J. Hazard. Mater.* **431**, 128602 (2022).

52. X. Feng, M. Ye, Y. Li, J. Zhou, B. Sun, Y. Zhu, L. Zhu, Potential sources and sediment-pore water partitioning behaviors of emerging per/polyfluoroalkyl substances in the South Yellow Sea. *J. Hazard. Mater.* **389**, 122124 (2020).

53. T. Han, J. Chen, K. Lin, X. He, S. Li, T. Xu, M. Xin, B. Wang, C. Liu, J. Wang, Spatial distribution, vertical profiles and transport of legacy and emerging per- and polyfluoroalkyl substances in the Indian Ocean. *J. Hazard. Mater.* **437**, 129264 (2022).

54. K. Hope, "Occurrence of major perfluoroalkyl substances (PFAS) in water samples from a transect of the Indian Ocean along the East African coast," thesis, Örebro University (2020).

55. H. Joerss, C. Apel, R. Ebinghaus, Emerging per- and polyfluoroalkyl substances (PFASs) in surface water and sediment of the North and Baltic Seas. *Sci. Total Environ.* **686**, 360–369 (2019).

56. H. Joerss, Z. Xie, C. C. Wagner, W.-J. von Appen, E. M. Sunderland, R. Ebinghaus, Transport of legacy perfluoroalkyl substances and the replacement compound HFPO-DA through the atlantic gateway to the Arctic Ocean—Is the arctic a sink or a source? *Environ. Sci. Technol.* **54**, 9958–9967 (2020).

57. L. Li, H. Zheng, T. Wang, M. Cai, P. Wang, Perfluoroalkyl acids in surface seawater from the North Pacific to the Arctic Ocean: Contamination, distribution and transportation. *Environ. Pollut.* **238**, 168–176 (2018).

58. D. d A. Miranda, J. Leonel, J. P. Benskin, J. Johansson, V. Hatje, Perfluoroalkyl substances in the western tropical atlantic ocean. *Environ. Sci. Technol.* **55**, 13749–13758 (2021).

59. M. A. Nguyen, K. Wiberg, E. Ribeli, S. Josefsson, M. Futter, J. Gustavsson, L. Ahrens, Spatial distribution and source tracing of per- and polyfluoroalkyl substances (PFASs) in surface water in Northern Europe. *Environ. Pollut.* **220**, 1438–1446 (2017).

60. J.-C. Picard, G. Munoz, S. Vo Duy, S. Sauvé, Longitudinal and vertical variations of waterborne emerging contaminants in the St. Lawrence Estuary and Gulf during winter conditions. *Sci. Total Environ.* **777**, 146073 (2021).

61. G. Shan, X. Qian, X. Chen, X. Feng, M. Cai, L. Yang, M. Chen, L. Zhu, S. Zhang, Legacy and emerging per- and poly-fluoroalkyl substances in surface seawater from northwestern Pacific to Southern Ocean: Evidences of current and historical release. *J. Hazard. Mater.* **411**, 125049 (2021).

62. Q. Wang, M. M. P. Tsui, Y. Ruan, H. Lin, Z. Zhao, J. P. H. Ku, H. Sun, P. K. S. Lam, Occurrence and distribution of per- and polyfluoroalkyl substances (PFASs) in the seawater and sediment of the South China sea coastal region. *Chemosphere* **231**, 468–477 (2019).

63. S.-K. Xiao, Q. Wu, C.-G. Pan, C. Yin, Y.-H. Wang, K.-F. Yu, Distribution, partitioning behavior and potential source of legacy and alternative per- and polyfluoroalkyl substances (PFASs) in water and sediments from a subtropical Gulf, South China Sea. *Environ. Res.* **201**, 111485 (2021).

64. E. Yamazaki, S. Taniyasu, Y. Ruan, Q. Wang, G. Petrick, T. Tanhua, T. Gamo, X. Wang, P. K. S. Lam, N. Yamashita, Vertical distribution of perfluoroalkyl substances in water columns around the Japan sea and the Mediterranean Sea. *Chemosphere* **231**, 487–494 (2019).

65. L. W. Y. Yeung, C. Dassuncao, S. Mabury, E. M. Sunderland, X. Zhang, R. Lohmann, Vertical profiles, sources, and transport of PFASs in the Arctic Ocean. *Environ. Sci. Technol.* **51**, 6735–6744 (2017).

66. X. Zhang, R. Lohmann, E. M. Sunderland, Poly- and perfluoroalkyl substances in seawater and plankton from the Northwestern Atlantic Margin. *Environ. Sci. Technol.* **53**, 12348–12356 (2019).

67. Z. Zhao, J. Tang, L. Mi, C. Tian, G. Zhong, G. Zhang, S. Wang, Q. Li, R. Ebinghaus, Z. Xie, H. Sun, Perfluoroalkyl and polyfluoroalkyl substances in the lower atmosphere and surface waters of the Chinese Bohai Sea, Yellow Sea, and Yangtze River estuary. *Sci. Total Environ.* **599–600**, 114–123 (2017).
